# The mechanisms and therapeutic advances of interactions between breast cancer and cardiovascular diseases

**DOI:** 10.3389/fphar.2026.1767098

**Published:** 2026-04-15

**Authors:** Meitong Liu, Chenchen Xu, Bo Dong

**Affiliations:** Department of Cardiology, Shandong Provincial Hospital Affiliated to Shandong First Medical University, Jinan, Shandong, China

**Keywords:** breast cancer, cardiovascular diseases, mechanism, diagnosis, therapy, prevention

## Abstract

Currently, breast cancer (BC) and cardiovascular diseases (CVD), as two major diseases that seriously threaten global public health, have become major public health problems that need to be urgently solved as their morbidity and mortality rates continue to rise. In recent years, with the continuous improvement of BC diagnosis and treatment, the overall survival of patients has been significantly prolonged, but CVD has gradually become one of the major non-oncological causes of death among BC survivors. It has been pointed out that there are multiple common mechanisms between BC and CVD at the pathophysiological level, including chronic inflammation, metabolic abnormalities, hormonal dysregulation and neuroendocrine system activation. This review summarizes the potential interactions between BC and CVD, the associated cardiotoxicity induced by cancer therapies, and the application of relevant biomarkers in diagnosis and risk assessment, with the aim of providing insights and reference for the comprehensive management of patients with coexisting BC and CVD.

## Introduction

In recent years, the increasingly close association between breast cancer (BC) and cardiovascular diseases (CVD) has attracted growing attention and become a major focus in clinical research. According to the latest data released by the International Agency for Research on Cancer (IARC) of the World Health Organization, BC, as the most common malignant tumor among women globally, has shown a continuously rising trend in incidence in recent years. In 2020 alone, there were approximately 2.26 million new cases of BC worldwide, surpassing lung cancer to become the most frequently diagnosed cancer globally (Sung et al., 2021). In China, there were an estimated 357,000 new cases and 75,000 deaths from BC in 2022, posing a serious threat to women’s health (Bray et al., 2024). BC and CVD share several common risk factors. Moreover, BC may exert detrimental effects on the initiation, progression, and adverse outcomes of CVD through mechanisms such as inflammation and oxidative stress, disruption of metabolic and hormonal balance, and treatment-induced cardiotoxicity (Mehta et al., 2018). Greenlee et al. (2022) reported that women with BC had higher rates of CVD, CVD-related mortality, and all-cause mortality compared to women without BC, with variations depending on risk factors and prior cancer treatments. This comorbidity severely impacts patient prognosis and quality of life. In this review, we systematically examine the relationship between BC and CVD, detail their shared risk factors, and explore the bidirectional mechanisms underlying the interaction between BC and CVD. In addition, we also address the cardiotoxic effects of BC therapies, and briefly outline integrated diagnosis and treatment strategies for patients with coexisting BC and CVD.

## The impact of breast cancer on cardiovascular diseases

### Common risk factors

Genetics, age, and sex are major non-modifiable risk factors shared by BC and CVD. Genetically, the association is mediated through shared susceptibility genes, as well as the familial transmission of metabolic abnormalities or hereditary traits linked to cardiovascular risk (Bai and Wu, 2024). Age is a major non-modifiable risk factor, and studies have demonstrated significant differences in the age distribution of BC patients between Eastern and Western populations. In Western countries such as the United States, the median age at BC onset is 62–64 years, with patients younger than 40 years accounting for only 4.9% of all cases. In contrast, in China and other East Asian countries, the median age at diagnosis is notably younger, approximately 45–49 years (Yap et al., 2019). As a core non-modifiable risk factor, sex exerts a profound and multifaceted effect on BC susceptibility, pathogenesis, and clinical outcomes. Male BC patients are usually diagnosed later, with a mean age of 65–70 years, compared to 55–60 years for female BC (Scomersi et al., 2021), with delayed diagnosis often leading to more advanced disease stages and poorer prognoses, partially due to lower awareness and underutilization of screening tools. For men, while baseline estrogen levels are significantly lower, conditions that disrupt androgen-estrogen balance—such as Klinefelter syndrome, obesity-induced aromatase activation, or chronic liver disease—markedly increase BC susceptibility (Sah et al., 2026). Notably, these same conditions are also linked to elevated CVD risk in men, including hypertension, dyslipidemia, and atherosclerosis, highlighting the shared sex-specific mechanistic pathways between BC and CVD.

In contrast, several modifiable risk factors play pivotal roles in the crosstalk between BC and CVD. Obesity is an important crossover risk factor between the two. It promotes peripheral adipose tissue aromatase activity and increases estrogen synthesis, thus facilitating estrogen receptor-positive BC, while also accelerating atherosclerosis development by inducing insulin resistance and systemic chronic inflammation. Unhealthy lifestyles, such as physical inactivity and poor dietary habits, easily lead to metabolic syndrome—characterized by hypertension, hyperglycemia, and dyslipidemia—which fosters a tumor-promoting microenvironment and impairs endothelial function, not only favors the formation of the tumor microenvironment but also induces vascular endothelial dysfunction, which promotes the progression of CVD. Smoking not only promotes the malignant transformation of breast epithelial cells but also facilitates atherosclerosis through nicotine-induced extracellular vesicle-miRNA (Pizzato et al., 2021; Wada et al., 2024). Alcohol consumption represents a double-edged sword. Moderate drinking has been associated with a 14%–25% reduction in the risk of coronary heart disease compared with abstinence. However, excessive alcohol intake leads to the production of acetaldehyde during metabolism, which induces DNA damage and elevates circulating estrogen levels, thereby significantly increasing the risk of BC (Rumgay et al., 2021). Long-term endogenous estrogen exposure or exogenous hormone therapy stimulates cell proliferation in BC and exerts a dual effect on CVD protective premenopause and potentially harmful postmenopause. Comprehensive interventions, such as weight management, regular exercise, and balanced nutrition, can simultaneously reduce the risk of both diseases. Given the multifaceted interplay of metabolic, inflammatory, and hormonal pathways, elucidating the underlying mechanisms is essential for accurate risk assessment and the development of integrated prevention and treatment strategies.

### Common pathogenic mechanisms

**FIGURE 1:**
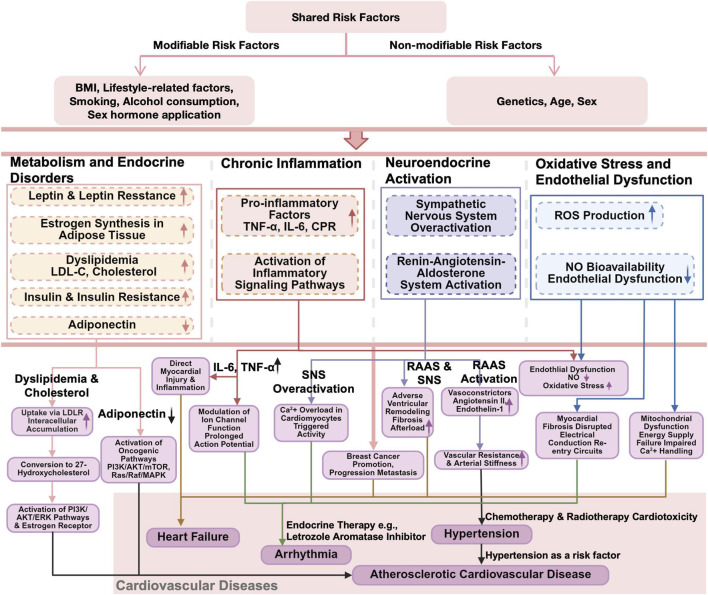
Breast cancer is pathophysiologically linked to various cardiovascular diseases, including coronary artery disease, heart failure, arrhythmia, and hypertension, through mechanisms involving metabolic and endocrine dysregulation, chronic inflammation, neuroendocrine activation, oxidative stress, and endothelial dysfunction.

Disruption of metabolic and endocrine homeostasis represents a core initiating background for cardiovascular vulnerability associated with BC, commonly manifested as reduced insulin sensitivity, reprogramming of lipid metabolism, and dysregulated estrogen signaling. These alterations impair the heart’s ability to match energy supply with demand, reduce metabolic reserve and stress adaptability, and thereby amplify the impact of chronic low-grade inflammation, neuroendocrine imbalance, and oxidative stress on the cardiovascular system. BC is frequently accompanied by persistent elevation of inflammatory mediators and sustained immune activation, generating a systemic inflammatory milieu of low intensity but long duration that promotes vascular inflammatory responses and myocardial interstitial remodeling. Concurrently, sustained activation of the sympathetic nervous system and the renin–angiotensin–aldosterone system (RAAS) further compromises cardiovascular adaptive capacity and, through interactions with metabolic and inflammatory disturbances, influences vascular tone regulation, myocardial structural stability, and cardiac electrophysiological balance. Oxidative stress and endothelial dysfunction represent parallel and interrelated pathological features in BC–associated cardiovascular vulnerability. Disruption of redox homeostasis together with impaired endothelial regulatory function can concurrently affect cardiomyocyte energy metabolism, vascular reactivity, and microcirculatory perfusion, thereby interacting with metabolic, inflammatory, and neuroendocrine abnormalities to form a multidimensional pathophysiological network. Collectively, these mechanisms coexist in a parallel and interactive manner, providing a unified framework for understanding BC–related cardiovascular injury and the heterogeneity of its clinical phenotypes, consistent with the parallel mechanistic structure illustrated in the accompanying figure (Figure 1).

### Breast cancer and atherosclerotic cardiovascular diseases

BC and atherosclerotic cardiovascular disease (ASCVD) share multiple pathogenic pathways. Adipose tissue secretes inflammatory factors such as tumor necrosis factor-α (TNF-α) and interleukin-6 (IL-6), which activate the Ras/Raf/MAPK and JAK/STAT signaling pathways, upregulating matrix metalloproteinase-9 (MMP-9) and promoting BC cell migration (Zhang and Liu, 2020; Kochumon et al., 2024). Obesity can cause fat accumulation and metabolic disorders. Leptin, a key adipokine, regulates the appetite and energy balance of the body mainly by acting on the brain and may protect against ASCVD by improving glucose-lipid metabolism (Pereira et al., 2021). However, leptin resistance is common in obese individuals. When leptin combines with IL-6, it can activate multiple signaling pathways, including Ras/Raf/MAPK, JAK/STAT, PI3K/Akt, and increase the expression of lysyl hydroxylase-2 (LH-2) or invasion-associated proteins, ultimately driving cancer cell survival, proliferation, and metastasis (Guo et al., 2012; He et al., 2018). In contrast, a lipocalin has been shown to significantly inhibit leptin-induced inflammation, proliferation, and survival of tumor cells, as well as vascular smooth muscle cell proliferation, migration, and foam cell transformation, while also reversing the leptin-associated anti-apoptotic effects (Delort et al., 2015; Habanjar et al., 2023), however, lipocalin levels tend to decrease in the obese state, which not only inhibits the AMPK pathway but also activates oncogenic signaling pathways such as Pl3K/AKT/mTOR,Ras/Raf/MAPK and other oncogenic signaling pathways (He et al., 2018), weakening its cardiometabolic and metabolic protective effects. Furthermore, the link between dyslipidemia and chemotherapy efficacy has also been demonstrated (Qu et al., 2020), In rapidly proliferating tumor cells, cholesterol is not only a precursor or steroid hormone synthesis but also a critical component of cell membrane formation. Emerging evidence suggests that elevated cholesterol, low - density lipoprotein cholesterol (LDL-C), and very low - density lipoprotein cholesterol (VLDL-C) levels may be an independent risk factor for BC (Nelson et al., 2014; Gu et al., 2022). The LDL receptor helps BC cells acquire more cholesterol by mediating endocytosis of LDL-C (Pires et al., 2012). In preclinical *in vitro* studies, Revilla et al. (2021) found that intracellular cholesterol accumulation can activate the Pl3K/Akt/ERK1/2 pathway and convert to 27-hydroxycholesterol, which can be used to synthesize cell membranes and signaling molecules, thereby promoting the proliferation of BC cells. Statins, as the most widely prescribed LDL-C-lowering agents, with robust evidence from large-scale randomized controlled trials (RCTs) supporting their efficacy in reducing ASCVD incidence. However, their specific anti-tumor mechanisms are primarily supported by experimental rather than prospective clinical data. For instance, using *in vivo* murine models, Li et al. (2024) found that lovastatin markedly suppresses paclitaxel-induced expression of programmed death-ligand 1 (PD-L1), inhibits tumor cell proliferation, and enhances the anti-tumor activity of CD8^+^ T cells. These findings suggest that therapeutic strategies targeting lipoprotein-mediated cholesterol uptake and storage may offer a promising approach to preventing or delaying the progression of hormone-dependent malignancies.

Body mass index (BMI) is strongly associated with ASCVD and BC, a higher BMI significantly increases the risk of both diseases. High BMI is one of the major contributors to the global burden of BC and shows a rising trend, particularly in low and middle-income countries, where middle-aged and older populations face a higher risk (Cai et al., 2025). The incidence of premenopausal BC has increased in high-income countries, which may be associated with prolonged hormonal exposure or the widespread implementation of screening programs. In contrast, the rising incidence of postmenopausal BC is predominantly observed in regions undergoing socioeconomic transition, such as Eastern Europe and Latin America, and is closely linked to the global obesity epidemic (Heer et al., 2020). BMI in postmenopausal women is an independent risk factor for BC (Renehan et al., 2008), and postmenopausal BMI is positively correlated with estrogen levels (Kakugawa et al., 2017). Studies have shown that estrogen biosynthesis in postmenopausal women is mainly found in adipose tissue, where adrenal androgens are converted to estrogen by aromatase (Liedtke et al., 2012), obesity-associated inflammatory mediators, such as prostaglandin E2 (PGE2), cyclooxygenase-2 (COX-2) (Harris, 2014; Engin et al., 2019; Gonçalves et al., 2021), increase local estrogen production in tumor cells and the surrounding stroma. Recent *in vitro* and animal models have indicated that the aromatase inhibitor letrozole may influence energy metabolism in cardiomyocytes through a non-estrogen-regulated pathway. Specifically, Letrozole downregulates the expression of fatty acid metabolism enzymes such as CPT1 and ACADVL while upregulating glucose uptake and glycolysis activity through key regulatory genes including HK2 and PKM2 (Heo et al., 2022). This finding suggests potential cardiovascular-related adverse reactions during letrozole treatment and also provides a research direction for the subsequent combination of metabolic regulatory drugs to protect the myocardium. However, since these conclusions rely entirely on preclinical models, further clinical validation is needed to determine whether these specific metabolic shifts directly translate to the cardiotoxicity observed in patients. Furthermore, a high BMI can lead to hyperinsulinemia and diabetes mellitus. Hyperinsulinemia may enhance estrogen-driven activation of the PI3K/AKT/mTOR and Ras/Raf/MAPK pathways by inducing phosphorylation modifications of ER or by strengthening its interactions with downstream signaling molecules. In addition, insulin can independently activate these pathways through binding to the insulin/IGF-1 receptor, thereby synergizing with estrogen signaling and amplifying proliferative effects via positive feedback (Kim and Scherer, 2021; Saini et al., 2013). Data from the UK Biobank cohort reveals that postmenopausal women with DM have a higher risk of BC than premenopausal women with DM. This may be due to hyperinsulinemia reducing levels of sex hormone-binding globulin (SHBG), increasing free estrogen, and enhancing the interaction between estrogen and its receptor on BC cells, thus promoting cell proliferation and survival (Samuel et al., 2018; Xiong et al., 2023). Given the inherent limitations of observational data, though, these underlying mechanisms still require further prospective clinical validation.

### Breast cancer and heart failure

Heart failure and BC share multiple common pathophysiological mechanisms. Compared with healthy individuals, IL-6 and TNF-α levels are significantly higher in patients with heart failure (Murphy et al., 2020; Alogna et al., 2023). Higher IL-6 levels are associated with increased severity of congestive heart failure (CHF) (Berger et al., 2024). A retrospective study by Liang et al. (2024) found that BC survivors have a higher risk of developing advanced CHF than the general population, with younger survivors showing particularly elevated risk. In terms of oxidative stress, HF patients often suffer from mitochondrial Ca^2^+ overload and reactive oxygen species (ROS) accumulation, leading to abnormal opening of the mitochondrial permeability transition pore (mPTP), collapse of the mitochondrial membrane potential (ΔΨm), disruption of ATP synthesis, and failure of the energy supply to cardiomyocytes (Zhou and Tian, 2018). The imbalance of mitochondrial energy metabolism directly impairs cardiac diastolic function. Similarly, tumor cells exhibit metabolic abnormalities and generate large amounts of ROS, which damage cardiomyocyte membranes and mitochondrial structures, eventually leading to myocardial energy metabolism disorders and functional decline. From a neuroendocrine regulation perspective, tumor-related chronic stress can activate the sympathetic nervous system (SNS) and the RAAS, resulting in vasoconstriction, sodium and water retention, and increased cardiac load, which can trigger or worsen HF and hypertension. Moreover, vascular endothelial growth factor (VEGF) from tumor cells may cause imbalanced myocardial microvascular angiogenesis and vascular structural abnormalities, leading to impaired myocardial perfusion. In a multicenter prospective cohort study involving 1,024 patients with acute decompensated heart failure, low levels of soluble VEGF receptor-2 (sVEGFR-2) were independently associated with cardiovascular and all-cause mortality in CHF patients, particularly in those with high levels of N-terminal pro-B-type natriuretic peptide (NT-proBNP) (Iguchi et al., 2021). Ultimately, these multifaceted processes are anchored in a shared immunological foundation. Persistent innate immune activation in both HF and cancer drives a chronic inflammatory state, evidenced by elevated TNF-α, IL-6, and interleukin-1β (IL-1β). In heart failure, macrophages orchestrate myocardial fibrosis by secreting profibrotic mediators like transforming growth factor-β (TGF-β) and platelet-derived growth factor (PDGF), which accelerate collagen deposition and structural remodeling. Parallelly, this inflammatory milieu remodels the tumor microenvironment to facilitate oncogenesis and progression (Zhou et al., 2016). In conclusion, these findings underscore a common pathological basis across metabolic, neuroendocrine, and immune dimensions.

The SNS plays a key role in heart failure and cardiovascular remodeling, also contributes significantly to BC development. Neurotransmitters such as norepinephrine released during its excitation can promote the proliferation of tumor cells through activation of adrenergic receptors on the surface of BC cells via signaling pathways such as MAPK (Jin et al., 2023), and also modulate the expression of angiogenic factors within the tumor microenvironment (Zhou et al., 2020a), and induce epithelial–mesenchymal transition (EMT) (Du et al., 2020), enhancing tumor cell migration and invasiveness. To evaluate the impact of chronic stress on tumor growth, Cui et al. (2019) utilized a chronic stress model in NOD/SCID mice, this study revealed that chronic stress activates β2-adrenergic receptors via adrenaline, significantly elevates the activity and expression of lactate dehydrogenase, promotes lactate production, creates an acidic microenvironment, activates the deubiquitinating enzyme USP28, and reduces the degradation of the oncogenic protein MYC, which upregulates the transcription factors SLUG and SOX9, endowing BC cells with stem-like phenotypes. Beyond this glycolytic shift, chronic stress also disrupts lipid metabolism to fuel tumor progression. Zhou Y. et al. (2024) found that ubiquitin-specific peptidase 22 (USP22) promotes epinephrine-induced lipolysis by stabilizing the adipose triglyceride lipase (ATGL) transcription factor FOXO1. Free fatty acids (FFAs) produced from lipolysis provide energy to tumor cells and, through activation of integrin-dependent pathways, enhance BC cell invasion and metastasis (Liu K. et al., 2024). Excessive FFAs uptake in the myocardium can lead to lipotoxicity and mitochondrial dysfunction, further exacerbating heart failure. While these preclinical findings elegantly delineate specific neuro-metabolic axes, the extent to which these stress-induced pathways act as simultaneous drivers of BC and HF in clinical settings requires further prospective validation.

RAAS, as an important humoral regulatory system in the body, maintains hemodynamic stability when activated acutely, while chronic and sustained activation promotes ventricular remodeling, aggravates cardiac load, and impairs myocardial function. Some studies have found that RAAS is also involved in cancer development. In normal breast tissues, RAAS activity mainly depends on the alternative pathway (ACE-2/Ang 1-7/MASR) (Martyniak and Tomasik, 2022), while in BC tissues, the classical pathway (ACE/Ang II/AT1R) predominates (Pawlonka et al., 2024). Ang ll primarily promotes BC progression, growth, EMT, angiogenesis, and metastasis mainly through AT1R (George et al., 2010). ACE inhibitors (ACEi) suppress the RAAS by limiting Ang II synthesis; this downregulation subsequently inhibits metastasis, proliferation, and angiogenesis within the tumor microenvironment (Arrieta et al., 2015; De Miranda et al., 2021). Angiotensin receptor blockers (ARBs), by blocking AT1R, similarly inhibit tumor progression and angiogenesis (Carlos-Escalante et al., 2021). Unrestrained tumor cells with RAAS activity respond to Ang II stimulation by expressing and secreting inflammatory cytokines, such as IL-8 and monocyte chemoattractant protein-1 (MCP-10), macrophage colony-stimulating factor (M-CSF), transcription factors (NF-κB, STAT), and VEGF (Rasha et al., 2019), which promote adhesion of cancer cells to the endothelium, hematopoiesis, angiogenesis, and facilitate tumor cell migration through degradation of the extracellular matrix, are directly involved in promoting a favorable environment for tumor progression Such findings further confirm the involvement of RAAS in BC pathogenesis (George et al., 2010).

### Breast cancer and arrhythmia

Inflammatory responses play a key role in the connection between BC and arrhythmia. Immune cells in the tumor microenvironment, such as tumor-associated macrophages (TAMs), T cells and fibroblasts, continuously releasing pro-inflammatory mediators like IL-6, TNF-α, and C-reactive protein (CRP), these factors not only promote tumor cell proliferation, invasion, and metastasis but also affect the heart through the circulation by altering the electrophysiological function of cardiomyocytes (Hu et al., 2015; Lazzerini et al., 2019), Inflammatory factors such as IL-6 and TNF-α have been found to modulate sodium, potassium, and calcium channels on cardiomyocyte membranes (London et al., 2003; Kawada et al., 2006; Aromolaran et al., 2024), altering action potential duration and myocardial repolarisation, increasing the risk of reentry and triggered activity, and thereby facilitating arrhythmias, particularly atrial fibrillation and ventricular ectopic rhythms. The potassium channel protein KCNK1 promotes the proliferation and metastasis of BC cells by activating lactate dehydrogenase A (LDHA) and up-regulating histone H3K18 lactate modification (Pan Kla) (Hou et al., 2024). The metabolic and endocrine mechanisms of BC similarly affect cardiomyocyte electrophysiology. Fluctuation or deficiency of estrogen levels can influence the expression of multiple channels on cardiomyocyte membranes, such as potassium channels (IKr, IKs) and calcium channels (Cav1.2α, NCX1) (Drici et al., 1996; Kurokawa et al., 2008; Papp et al., 2017), This can prolong ventricular repolarization, increase the QT interval, and heighten the risk of ventricular arrhythmias. These cardiotoxic effects are especially relevant for patients with underlying CVD.

BC can affect cardiac microcirculation and myocardial tissue structure through various mechanisms. Tumor-related inflammatory cytokines, VEGF, and metabolic abnormalities can act on the cardiac microvascular system through the blood circulation, resulting in uneven myocardial capillary perfusion, vascular endothelial dysfunction, and microvascular remodeling. These changes may cause localized ischemia, cardiomyocyte damage and interstitial fibrosis, and interfere with the normal conduction of electrical signals. Chronic ischemia from microcirculatory impairment can result in altered cardiomyocyte structure and function, including decreased function of sarcoplasmic reticulum calcium pumps, restricted mitochondrial energy metabolism, and decreased expression of intercellular gap junction proteins like connexin 43 (Cx43) (Kho, 2023; Hinton et al., 2024; Rusiecka et al., 2020). These alterations will slow conduction velocity, delay regional repolarization, and promote conduction block, thereby facilitating arrhythmias—especially ventricular arrhythmias and reentrant circuits. Excessive deposition of collagen in the myocardial interstitium is also one of the manifestations of BC-induced cardiac fibrosis, which not only affects myocardial compliance and contractility but also disrupts synchronized electrical signal propagation in the myocardium. One study has reported that have reported that myocardial T1/T2-weighted magnetic resonance imaging (MRI) in patients with BC demonstrates myocardial tissue edema and an elevated interstitial volume fraction, suggesting subclinical myocardial involvement even in the absence of overt symptoms in BC people (Cheng et al., 2023).

The autonomic nervous system (ANS) essential for regulating cardiac electrical activity and rhythm stability, can also be affected by BC through multiple pathways, thereby disrupting cardiac rhythmic homeostasis, which can be manifested as heightened sympathetic activity (Szpunar et al., 2016; Silva et al., 2022). decreased heart rate variability (HRV) (Majerova et al., 2022). Reduced HRV reflects autonomic imbalance and has been linked to increased tumor cell migration and angiogenesis. Cancer-related psychological stress, pain, sleep disturbances, and tumor-secreted signaling molecules—such as neurotrophic factors and catecholamines—can all activate the hypothalamic–pituitary–adrenal (HPA) axis and further stimulate the SNS, placing the heart under sustained stress (Thornton et al., 2010; Minkel et al., 2014; Herman et al., 2016; Tausk, 2023). Sympathetic overactivation can lead to intracellular calcium overload in cardiomyocytes, increasing the probability of early and delayed afterdepolarizations, and triggering arrhythmias (Zhang D. et al., 2021), In acute ischemia, norepinephrine and epinephrine levels significantly rise in atrial and ventricular tissue, directly promoting atrial fibrillation (Zhou et al., 2016). Furthermore, tumor-associated inflammatory factors can impair sympathetic nerve terminal function, worsening autonomic imbalance. Therefore, autonomic dysregulation may be a key mediator linking BC and arrhythmias.

### Breast cancer and hypertension

Hypertension is not only a major risk factor for CVD. In BC, A retrospective clinical analysis involving individuals previously treated with anthracyclines and/or trastuzumab revealed significant racial disparities in cardiovascular sequelae. Specifically, Black BC survivors exhibited a markedly higher incidence of hypertension compared to their White counterparts. This difference remained significant even after adjusting for confounding factors such as age, BMI, and smoking history (Williams et al., 2020). Beyond its role as a comorbidity, hypertension directly influences the tumor microenvironment. Mechanistically, hypertension-induced hemodynamic stress triggers chronic endothelial injury and vascular remodeling. This vascular dysfunction facilitates tumor cell adhesion to the endothelium, simplifies their transendothelial migration, and ultimately promotes distant metastasis (Van Dorst et al., 2021). Furthermore, the systemic activation of the RAAS associated with hypertension acts as a potent oncogenic stimulus. Elevated levels of Angiotensin II can disrupt the normal physiological signaling of breast epithelial cells, promoting aberrant proliferation and establishing a pro-tumorigenic niche (Ishikane and Takahashi-Yanaga, 2018).

The chronic inflammatory state of the BC microenvironment can directly affect vascular homeostasis. Tumor cells continuously release various pro-inflammatory factors, and upregulate the expression of endothelial adhesion molecules, inhibit endothelial nitric oxide synthase (eNOS) activity, reduce nitric oxide production, and promote the release of vasoconstrictive substances, such as Ang II and endothelin-1, thereby contributing to elevated blood pressure (Versmissen et al., 2019; Dobbin et al., 2021), BC cells and their secreted extracellular vesicles can also affect distant vasculature through blood circulation, inducing smooth muscle cell proliferation, endothelial dysfunction, and arteriolar fibrosis. In particular, TGF-β and VEGF may promote vasoconstriction and arterial stiffening in the cardiovascular system (Matsumoto and Mugishima, 2006; Chen et al., 2019), These changes ultimately increase vascular resistance and reduce compliance, laying the foundation for hypertension.

## Effect of breast cancer treatment on cardiovascular diseases

Although advancements in BC therapies have remarkably extended patient survival, the diverse treatment landscape—including chemotherapy, targeted therapy, immunotherapy, and radiotherapy—imposes varying degrees of cardiovascular toxicity. These therapeutic modalities pose a major health threat to long-term survivors, potentially inducing high-mortality cardiovascular events. As summarized in Table 1, we systematically categorize these toxicities by treatment type and provide a comprehensive overview based on the latest research findings in the following sections.

**TABLE 1:** Cardiotoxicity Classification of anti-cancer drugs.

Classification	Drugs class	Cardiotoxicity mechanism	Diseases	Clinical studies	Preclinical studies
Chemotherapy	Anthracyclines Doxorubicin Epirubicin Daunorubicin Idarubicin	1. Oxidative Stress: Mitochondrial ROS burst leads to cardiolipin peroxidation 2. Calcium Homeostasis Dysregulation: Disturbed calcium handling and mitochondrial calcium overload promote cardiomyocyte dysfunction and death 3. Ferroptosis: Iron metabolism dysregulation triggers lethal lipid peroxidation 4. Proteostasis: Inhibition of autophagic flux leads to accumulation of damaged mitochondria 5. Type I Injury (Irreversible): Binding to TOPIIβ causes DNA double-strand breaks and transcriptional interference	Type I Injury (Irreversible) Cumulative heart failure Cardiomyopathy	Carreira et al. (2025) Lim et al. (2025) Poovorawan et al. (2025)	Refer to Table 2
​	Taxanes Paclitaxel Docetaxel	1. Microtubule Hyper-stabilization: Immobilizes the tubulin cytoskeleton, disrupting the membrane localization and trafficking of ion channels 2. Conduction Interference: Directly inhibits spontaneous depolarization and signal propagation within the AV node, inducing bradycardia 3. Pharmacokinetic Interaction: Inhibits P-glycoprotein and CYP450, delaying anthracycline clearance and elevating cardiotoxic metabolites like doxorubicinol	Conduction Block: Bradycardia Atrioventricular block	Kézdi et al. (2025)	Mann et al. (2020)
​	Alkylating Agents Cyclophosphamide Carboplatin Cisplatin	1. Endothelial Barrier Disruption: High-Dose Cyclophosphamide direct toxic injury to the capillary endothelium and endocardium by acrolein metabolites, triggering microvascular hemorrhage, severe interstitial edema, and acute cardiomyocyte necrosis	Myocardial Ischemia pericarditis	Tang et al. (2025)	Marchetti et al. (2025) Elsayed et al. (2025)
​	Antimetabolites 5-fluorouracil Capecitabine Gemcitabine Methotrexate Pemetrexed	1. Nucleotide pool depletion: Disrupts *de novo* purine/pyrimidine synthesis 2. Impaired nucleic acid synthesis: Blocks DNA/RNA production and repair in cardiomyocytes 3. Energy metabolic disturbance: Reduces ATP generation and impairs contractile function 4. Coronary vasospasm: Fluoropyrimidine-induced vascular dysfunction triggers myocardial ischemia	Coronary vasospasm Angina pectoris	Sapapsap et al. (2024) Zafar et al. (2022)	Ibrahim et al. (2023) Li et al. (2023)
Targeted therapy	Anti-HER2 agents Antibodies Trastuzumab Pertuzumab	1. Synergistic Dimerization Blockade: Trastuzumab binds to Domain IV to inhibit ligand-independent homodimerization (HER2-HER2), while Pertuzumab binds to Domain II to physically obstruct ligand-induced heterodimerization (HER2-HER3/4), achieving comprehensive signaling blockade 2. Multi-pathway Signaling Sabotage: Interruption of NRG1-ErbB, PI3K/Akt, and MAPK pathways, paralyzing cardiomyocyte repair and triggering inflammatory response 3. Type-II (Reversible) Dysfunction: Unlike the permanent structural damage caused by anthracyclines, HER2 inhibition leads to dose-independent, non-necrotic, and typically reversible functional decline that often recovers upon drug cessation	Asymptomatic LVEF Decline Heart Failure	Xia et al. (2025) Akbalaeva et al. (2025)	Mikami and Adachi-Akahane (2026) Zhu et al. (2025a) Diwanji et al. (2021)
​	Anti-HER2 antibody-drug conjugates Trastuzumab emtansine Trastuzumab deruxtecan	1. Toxicity Duality: While minor toxicity is target-mediated, >99% of systemic damage is driven by non-targeted uptake; the chemical nature of the payload—rather than the antibody target—is the primary determinant of clinical tolerance and toxicity profiles 2. Off-target and Linker Dynamics: Non-specific endocytosis is fueled by physicochemical properties (high DAR/hydrophobicity) and Fc-mediated internalization; maintaining linker stability is critical to balancing the “bystander effect” with systemic drug leakage 3. Immune Modulation Cascade: By inducing ICD and directly maturing DCs, ADCs convert direct cytotoxicity into a systemic immune response, utilizing Fc-mediated effects (ADCC/ADCP) to remodel the immunosuppressive tumor microenvironment	Heart failure, Arrhythmias	Schettini et al. (2025) Long et al. (2024)	Nguyen et al. (2023) Aoyama et al. (2022)
​	CDK4/6 inhibitors Palbociclib Ribociclib Abemaciclib	1. Potassium Channel Blockade: hERG channel interference 2. Vascular Endothelial Dysfunction	QT Prolongation, VTE Hypertension	Hoo et al. (2026) Li et al. (2025b) Cicini et al. (2022)	Tao et al. (2017)
​	PARP inhibitors Olaparib Niraparib Talazoparib	1. Endothelial Dysfunction and Systemic Hypertension 2. Mitochondrial Bioenergetic Impairment	Hypertension Arrhythmias	Spring et al. (2022)	Kim et al. (2025) Chen et al. (2025b)
​	PI3K/mTOR inhibitors Alpelisib Everolimus	1. Endothelial Dysfunction: Blockade of the AKT/eNOS axis reduces NO production, causing impaired vasodilation and systemic hypertension 2. Systemic Metabolic Remodeling: Disruption of insulin signaling triggers hyperglycemia and dyslipidemia, escalating oxidative damage and cardiac afterload 3. Bioenergetic and Autophagy Failure: Suppression of mTOR-mediated autophagy and mitochondrial ATP production leads to proteotoxicity and cardiomyocyte apoptosis	Hypertension Dyslipidemia	Arena et al. (2021)	Browne et al. (2024) Ariaans et al. (2017)
Endocrine therapy	SERMs Tamoxifen Toremifene Raloxifene	1. Prothrombotic Effects: Tamoxifen and Raloxifene enhance coagulation factor expression, reduce anticoagulant activity, and impair fibrinolysis, leading to increased risk of venous thromboembolism 2. Context-Dependent Lipid Modulation: SERMs generally reduce LDL cholesterol through hepatic ERα activation but may increase triglyceride levels in susceptible individuals, resulting in a mixed impact on atherosclerotic risk 3. Electrophysiological Disturbance: Modulation of cardiac ion channels and mitochondrial signaling may increase susceptibility to QT prolongation and arrhythmias	VTE Stroke Pulmonary embolism Hypertriglyceridemia-Related Complications Arrhythmia	Huang et al. (2025b) Cheang et al. (2025)	Xie et al. (2023) Chen et al. (2023b)
​	AIs Anastrozole Letrozole Exemestane ·	1. Dyslipidemia increased LDL and altered lipid profile 2. Endothelial dysfunction reduced nitric oxide and vascular inflammation	Ischemic heart disease Heart failure	Sekmek et al. (2025) Lund et al. (2024)	Mukherjee et al. (2022)
​	GnRH Agonists Goserelin Leuprorelin Triptorelin	1. Metabolic dysregulation:Estrogen deprivation induced metabolic remodeling characterized by insulin resistance dyslipidemia weight gain and metabolic syndrome	Metabolic syndrome; Metabolic syndrome–related cardiovascular events	Manfrini et al. (2025) Cicione et al. (2023) Bergami et al. (2023)	NA
​	SERDs Fulvestrant	1. Proarrhythmic Potential:Estrogen receptor degradation may affect cardiac ion channels leading to QT	NA	Escrivá-de-Romani et al. (2025)	Basu et al. (2026)
Immunotherapy	PD-1/L1 Pembrolizumab Nivolumab Atezolizumab Durvalumab	1. Immune-mediated Injury:PD-1/PD-L1 blockade overactivates T cells, triggering an attack on cardiomyocytes expressing shared or homologous antigens. This immune-mediated response leads to extensive myocardial inflammation and myofibril destruction 2. Inflammatory Cytokine Storm: Activation of T cells/fibroblasts triggering a pro-inflammatory cytokine cascade (e.g., IFN-γ, TNF-α, CXCL9/10) and immune cell infiltration, inducing myocardial inflammation and injury 3. Autoantibody Production: The treatment may trigger the production of autoantibodies against cardiac antigens, activating the complement system and recruiting additional immune cells to exacerbate cardiomyocyte injury	Immune-Related Myocarditis; Atrial Fibrillation Disorders; Pericardial Diseases Heart Failure	El Fakih et al. (2026) Chang (2025) Suero-Abreu et al. (2025)	You et al. (2026) Gao et al. (2026) Wang et al. (2025e) Xuan et al. (2025) Zhang et al. (2025c) Huang et al. (2025c) Jovanovic et al. (2025)
​	CTLA-4 Inhibitors Ipilimumab Tremelimumab	1. Threshold-Breach Synergy:The mechanisms of CTLA-4 and PD-1 pathways are functionally interdependent. Dual blockade disrupts the co-inhibitory crosstalk between the T-cell priming phase (CTLA-4) and the effector phase (PD-1), causing a synergistic collapse of immune tolerance. This interaction permits T-cell activation to exceed critical safety thresholds, leading to rapid and aggressive myocardial auto-reactivity	Immune-Related Myocarditis; ST elevations; Arrhythmia Heart Failure;	Xiao et al. (2023) Mahmood et al. (2018)	Wei et al. (2021)
​	LAG-3 Inhibitors Relatlimab Favezelimab Eftilagimod alpha	1. CXCR6/CXCL16-Driven Myocardial Infiltration:CD8^+^ CXCR6^+^ T-cell recruitment via the CXCL16-CXCR6 axis driving terminal injury, potentially initiated by LAG-3/MHC-II disruption on CD4^+^ T cells	Immune-Related Myocarditis;	Zhang et al. (2025a)	Munir et al. (2026) Lauder et al. (2026) Zhang et al. (2023a) Ozasa et al. (2025)
Radiation Therapy	NA	1. Microvascular Rarefaction: Radiation-induced DNA damage to endothelial cells triggers premature senescence and apoptosis, leading to a progressive reduction in myocardial capillary density and compensatory interstitial fibrosis 2. Accelerated Coronary Atherosclerosis:Radiation-induced oxidative stress promotes the formation of unstable atherosclerotic plaques within the major coronary arteries (particularly the LAD), significantly increasing the risk of premature myocardial infarction 3. Valvular and Pericardial Fibrosis:Chronic activation of TGF-β signaling pathways leads to the thickening and calcification of heart valves and the pericardium, resulting in restrictive cardiomyopathy and valvular dysfunction	Ischemic Heart Disease; Myocardial Fibrosis; Diastolic Dysfunction; Valvular Heart Disease	Belardo et al. (2025) Perman et al. (2024) Cao et al. (2025a)	Baselet et al. (2019)

Cytochrome P450, CYP450; Human Epidermal Growth Factor Receptor 2, HER2; Cyclin-Dependent Kinase 4/6, CDK4/6; Drug-to-Antibody Ratio, DAR; dendritic cells, DCs; Antibody-Dependent Cellular Cytotoxicity, ADCC; Antibody-Dependent Cellular Phagocytosis, ADCP; venous thromboembolism, VTE; Poly (ADP-Ribose) Polymerase, PARP; Human Ether-à-go-go-Related Gene, hERG; selective estrogen receptor modulators, SERMs; Estrogen Receptor Alpha, ERα; Aromatase Inhibitors, AIs; Gonadotropin-Releasing Hormone, GnRH; selective estrogen receptor degraders, SERDs; Programmed Cell Death Protein 1/Programmed Cell Death Ligand 1, PD-1/L1; Cytotoxic T-Lymphocyte-Associated Protein 4, CTLA-4; Lymphocyte-Activation Gene 3, LAG-3.

### Chemotherapy

Chemotherapy encompasses a broad spectrum of antineoplastic agents with distinct mechanisms of action and heterogeneous cardiotoxic profiles. While various chemotherapeutic drugs have been implicated in cardiovascular injury, anthracyclines remain the most extensively studied class and serve as a prototypical model for elucidating the molecular mechanisms underlying chemotherapy-induced cardiotoxicity. Doxorubicin-induced cardiotoxicity (DIC) represents a sophisticated failure of the systemic multicellular interactome rather than a localized cardiac insult, driven by multiple interconnected pathological processes. The pathogenic chain is initiated by an upstream gatekeeping dysregulation involving the immune-endothelial axis. IFN-γ acts as a regulator by inducing transcriptomic reprogramming in cardiac microvascular endothelial cells, compromising their role as a selective physical and biochemical barrier (Ji et al., 2025). This reprogramming, coupled with DOX-induced VEGFR2 downregulation and endothelial senescence, facilitates the excessive trans-endothelial accumulation of DOX into the myocardial interstitium while simultaneously triggering microvascular rarefaction (Graziani et al., 2022). Upon infiltrating the cardiac parenchyma, DOX activates a synchronized stress response across the cardiac triad—comprising cardiomyocytes, Endothelial Cells, and fibroblasts—centrally coordinated by a molecular hub involving RHO GTPases (RAC1 and CDC42) (Kücük et al., 2024).

Among these mechanisms, the redox cycling of doxorubicin and the consequent excessive generation of reactive ROS are widely recognized as key initiating events. Sustained ROS accumulation directly mediates DNA damage and structural abnormalities of mitochondria, while this intrinsic cellular injury is significantly amplified through localized neuroendocrine-immune crosstalk. Infiltrating macrophages establish a pro-inflammatory feedback loop by synthesizing and releasing catecholamines. These mediators stimulate β-adrenergic receptors (β-AR) on cardiomyocytes (Gambardella et al., 2023), subsequently activating the p53 signaling axis, which culminates in mitochondrial dysfunction and disruption of Ca^2+^ homeostasis. Parallelly, DOX reprograms cardiac fibroblasts toward a “pro-adhesive” phenotype by upregulating (intercellular adhesion molecule 1) ICAM-1 expression via the (signal transducer and activator of transcription 1–interferon regulatory factor 1) STAT1/IRF1 axis. This molecular switch promotes the recruitment and anchoring of circulating CD8^+^ T cells through lymphocyte function-associated antigen 1 (LFA-1)/ICAM-1 specific binding. Crucially, the direct physical contact between endothelial cells-anchored fibroblasts and CD8^+^ T cells triggers a non-canonical, antigen-independent degranulation of cytotoxic T lymphocytes (CTLs). This contact-dependent process drives the release of Granzyme B, which acts directly on neighboring fibroblasts to induce their phenotypic transition into profibrotic myofibroblasts (α-smooth muscle actin, α-SMA+). The resulting excessive deposition of extracellular matrix (ECM) proteins, such as Collagen I and III, leads to increased myocardial stiffness, reduced mechanical compliance, and ultimately, irreversible left ventricular systolic dysfunction (Bayer et al., 2024). Collectively, these dynamic multicellular interactions create a pro-toxic milieu that promotes diverse alterations in cardiomyocyte fate, including apoptosis, autophagy, necrosis, pyroptosis, senescence, and ferroptosis.

Owing to the high complexity and extensive crosstalk among multiple pathogenic processes in DIC, mechanism-based intervention strategies targeting critical molecular pathways and signaling networks have attracted increasing attention. Emerging evidence suggests that, beyond pharmacological agents and natural compounds, diverse molecular regulators and pathway-modulating approaches can affect these pathophysiological mechanisms and thereby modulate DOX-induced myocardial injury. In this section, we provide a systematic overview of recently identified intervention strategies, molecular targets, and related mechanisms in DIC (Table 2), and classify them based on the key signaling pathways involved.

**TABLE 2:** Recent advances in doxorubicin-induced cell injury mechanisms.

Author	Target	Mechanistic pathway	Outcome
Autophagy			
Xin et al. (2025)	Met	Met-GCN2→PINK1-Parkin↑/ATF4↑→restores autophagic flux	Attenuates
Xu et al. (2025)	overexpression hnRNPK	overexpression hnRNPK/Inhibit its cytoplasmic translocation→PINK1/Parkin↑→DOX-induced lipid accumulation↓	Attenuates
Jiang et al. (2025)	HINT2	Hint2 knockout→TFAM↓/OXPHOS complex I↓/NAD+/NADH↓→ lysosomal dysfunction↓	Attenuates
Momen et al. (2025)	Rosuvastatin	Rosuvastatin→SIRT1/FOXO1↑→ PINK1/parkin↑→restores autophagic flux	Attenuates
Hu et al. (2025)	Sacubitril/valsartan	Sacubitril/valsartan→AMPKα/mTORC1↑→ Enhance autophagic flux	Attenuates
Chen et al. (2025c)	20-Deoxyingenol	20-Deoxyingenol→UCHL3↑/TEEB↑→restores autophagic flux	Attenuates
Chang et al. (2025)	FoxO3	FoxO3 overexpression →LC3B↑/p62↓/mTOR↓/ROS↓→restores autophagic flux	Attenuates
Bian et al. (2025)	Quercetin	Quercetin→ EGFR Activation→restores autophagic flux	Attenuates
Zeng et al. (2024)	Vericiguat	Vericiguat→sGC/cGMP/PRKG1→PINK1/PARKIN↑→mtDNA↓→STING-IRF3↓→DIC↓	Attenuates
Lin et al. (2024b)	Dihydroartemisinin	Dihydroartemisinin→Keap1↓/Nrf2↑→LC3-II↑→CTSB/CTSD↑→restores autophagic flux	Attenuates
He et al. (2024)	FUNDC1	FUNDC1 overexpression → repairs MERCs →ATG5-ATG12/ATG16L1→restores autophagic flux	Attenuates
Chen et al. (2022)	Elabela	Ela→TFEB↑→restores autophagic flux	Attenuates
Pan et al. (2021)	Irisin	Irisin→UCP2↑→ROS-NF-κB-Snail↓→EndMT↓/restores autophagic flux	Attenuates
Apoptosis			
Sun et al. (2026)	PTN	PTN-SIRT1→AMPK/PGC1α↑→ROS↓/energy reprogramming↑	Attenuates
Li et al. (2026)	chromatin-immune	Warburg effect↑→Lactate↑→Lactyl-CoA↑→H3K18la/H3K9la/H3K14la↑→Transcriptional↑ →Oncogenes↑/Immune regulation↑→PD-L1↑/NLRP3↑/M2 MФ↑→immune escape↑	Promotes
Hu et al. (2026)	Serinc2	overexpresssion Serinc2→STAT3↑→Opa1/Mfn2↑→ROS↓/ATP↑→apoptosis↓	Attenuates
Zhao et al. (2025c)	ROS-responsive TPP–TS IIA micelle	DIC→ROS↑→ROS-responsive TPP–TS IIA micelle→TS IIA→ROS↓/mt damage↓→apoptosis↓	Attenuates
Zhao et al. (2025b)	aerobic exercise	Aerobic exercise→AMPK↑→PI3K/AKT↑→apoptosis↓	Attenuates
Zhao et al. (2025a)	DDX3X	DDX3X-MAVS→G3BP1-SGs↑→apoptosis↓	Attenuates
Yang et al. (2025a)	Tirzepatide	Tirzepatide→ER stress↓→HRD1-Nrf2 degradation↓→Nrf2 protein↑→HO-1/SOD/CAT↑→ROS↓→apoptosis↓	Attenuates
Ma et al. (2025a)	CYP2E1	DIC → CYP2E1 ↑ → L-OPA1/S-OPA1 ↓→ Cytc↑/Caspase-3↑/ROS ↑→ apoptosis↑	Promotes
Wu et al. (2025a)	Peptide MODICA	sORF-MODICA→VDAC3 oligomerization↓→Maintaining mitochondrial outer membrane integrity →apoptosis↓	Attenuates
Xue et al. (2025a)	gasdermin E	gasdermin E→mtDNA↑→STING/NF-κB↑→CCL2/CCR2↑→DIC↑	Promotes
Liu et al. (2025b)	Xinmailong	Xinmailong→MDH2↑→ATP↑→apoptosis↓	Attenuates
Ibrahim et al. (2025)	Methylene Blue	Methylene Blue→KEAP1↓/NRF2↑/GPX4↑→p53↓/Caspase-3↓→apoptosis↓	Attenuates
Wang et al. (2025f)	Empagliflozin	Empagliflozin→AMPK/SIRT-1/PGC-1α↑,PI3K/AKT/Nrf2↑→RIPK1↓→ROS↓→apoptosis↓	Attenuates
Feng et al. (2025)	CDC20	overexpression CDC20→ ubiquitination of CCDC69↓→ apoptosis↓	Attenuates
Wu et al. (2025b)	CCR9/CCL25	DOX→CCR9↑/CCL25↑→AMPK↓/CYP1A1↑→ROS↑/fibrosis↑/apoptosis↑	Promotes
Wei et al. (2025)	Trilobatin	Copper dyshomeostasis→FDX1-mediated protein lipoylation↑→ TCA cycle enzymes↓→cuproptosis↑ Trilobatin→FOX1↓→cuproptosis↓	Attenuates
Wang et al. (2025b)	miR-146b	overexpression miR→146b-HIF-1α↓→apoptosis↓	Attenuates
Tiwari et al. (2025)	Ast	Ast→ p-laminA/C↓→ DNMT1↓→ GATA-4/Bcl-xL↑→ apoptosis↓	Attenuates
She et al. (2025)	HEY2	DOX→HEY2↑→HDAC1→ Ppargc/Cpt↓→ETC-ATP↓	Promotes
Pan et al. (2025)	Tetrahedral Framework Nucleic Acids	Tetrahedral Framework Nucleic Acids→AKT/p53→apoptosis↓	Attenuates
Xie et al. (2024)	ADAM17	DOX↑→ ADAM17↑ → TNFα↑ → TRAF3/TAK1/MAPK↑ →apoptosis↑	Promotes
Choksey et al. (2024)	AICAR	AICAR→AMPK/PGC1α/β↑→restore lipid metabolism	Attenuates
Pyroptosis			
Rao et al. (2026)	Chicory Extract	Chicory Extract→UCP2↑/NLRP3↓→ROS↓→pyroptosis↓	Attenuates
Xue et al. (2025a)	gasdermin E	GSDME→STING/NF-κB→CCL2/CCR2→ TNF-α, IL-6, IL-1β↑→pyroptosis↑	Promotes
Ma et al. (2025c)	miR-216a-5p	miR-216a-5p → ITCH ↓ → TXNIP/NLRP3/Caspase-1↑→ pyroptosis↑	Promotes
Tian et al. (2024b)	Astragaloside IV	Astragaloside IV→Sirt1↑→NLRP3↓→pyroptosis↓	Attenuates
Zhang et al. (2021b)	MCC950	MCC950→NLRP3↓→caspase-1/GSDMD↓→IL-1β,IL-18↓→pyroptosis↓	Attenuates
Zhang et al. (2023b)	Mel	Mel→Sirt1/Nrf2↑→pyroptosis↓	Attenuates
Meng et al. (2019)	RNA TINCR	DOX→H3K27ac↑→TINCR↑ →TINCR-IGF2BP1→NLRP3 mRNA↑→NLRP3↑→pyroptosis↑	Promotes
Senescence			
Fan et al. (2025)	Rosmarinic acid	Rosmarinic acid→14-3-3γ/FoxO1→SA-β-gal↓→p16/p21↓→Il-6, Il-1β, Tnf-α↓→senescence↓	Attenuates
Xiong et al. (2025)	DNase1	overexpression DNase1→mtDNA/cGAS/STING↓→senescence↓	Attenuates
Shen et al. (2025)	FGF13	FGF13→Cav1/p53/p21→senescence↑	Promotes
Ferroptosis			
Yang et al. (2025c)	Xin-Ji-Er-Kang	Xin-Ji-Er-Kang→Nrf2-GPX4/FTH1→ ferroptosis↓	Attenuates
Ke et al. (2025)	NEDD4L	DOX→NEDD4L↑→GPX4↓→ferroptosis↑/Apoptosis↑	Promotes
Yang et al. (2025b)	Herbacetin	Herbacetin/ACSL4→ ferroptosis↓	Attenuates
Ma et al. (2025b)	Trim65	Trim65 → p53↓ → ferroptosis↓	Attenuates
Han et al. (2025)	sEVs	miR-338-3p-sEVs→anti-ferroptotic genes CP/SLC7A11/GPX4↓	Attenuates
Huang et al. (2025a)	Lycopene	Lycopene→ Nrf2↑→GPX4,HO-1↑→ ferroptosis↓ Lycopene→MFN1↑/DRP1↓→ Mitochondrial function restoration↑	Attenuates
Zhang et al. (2025b)	Kaempferol	Kaempferol→ NRF2/SLC7A11/GPX4→ferroptosis↓	Attenuates
Chen et al. (2025e)	EBBP	EBBP→ PERK/eIF2α/ATF4 → SLC7A11/GPX4↑→ ferroptosis↓	Attenuates
Fang et al. (2025)	Alox5	Alox5→P53/SLC7A11→ ferroptosis↓	Attenuates
Lin et al. (2024a)	Sarmentosin	Sarmentosin→p62/Keap1/Nrf2→ferroptosis↓	Attenuates
Shi et al. (2025)	AIG1	AIG1→Pirh2/p53→ferroptosis↓	Attenuates
Guo et al. (2025)	5-Oxoproline	GGCT↓←5-Oxoproline→OPLAH↑→ferroptosis↓	Attenuates
Others			
Wan et al. (2025)	Slc25a49	Slc25a49→G6P/AP-1/Sln↓→DIC↓	Attenuates
Thonusin et al. (2025)	α7nAChR/mAChR	α7nAChR/mAChR agonist→reduced cardiac glycolysis and restored fat utilization, succinate oxidation, and ATP production	Attenuates
Kubeš et al. (2025)	Topobexin	Topobexin-TOPIIATPase→ ATP↑	Attenuates
Lin et al. (2025)	Akkermansia muciniphila	Akkermansia muciniphila→PPARα/PGC1α→mitochondrial function↑	Attenuates
Li et al. (2025c)	KDM4C	Inhibition KDM4C→CTSL↑→histone H3↑→unfold protein response/IL2-STAT5/p53/TGF-β/TNF-α/NF-κB→cancer cells↓	Attenuates
Bao et al. (2024)	PICALM	DOX→PICALM↑→Aβ40↑→cell membranes↑→DIC↑	Promotes

#### The pathogenic network of mitochondrial collapse, pyroptosis, and sterile inflammation

Mitochondrial dysfunction acts as a fundamental determinant of DIC, manifesting as a critical convergence of profound structural damage, bioenergetic collapse, and severe oxidative stress. Driven by its high mitochondrial affinity, the quinone moiety of DOX undergoes continuous redox cycling catalyzed by cytosolic NADPH-dependent reductases, leading to a massive accumulation of superoxide anions. Because cardiac tissue inherently possesses exceptionally low baseline levels of antioxidant enzymes such as superoxide dismutase and catalase, it is acutely susceptible to reactive oxygen species generation and oxidative stress accumulation. Within the myocardium, which inherently possesses low levels of antioxidant enzymes, these superoxides interact with mitochondrial iron to drive the Fenton reaction, yielding highly reactive hydroxyl radicals. Concurrently, they react with upregulated nitric oxide synthase-derived nitric oxide to generate highly cytotoxic peroxynitrite. This profound nitro-oxidative stress rapidly overwhelms endogenous antioxidant defenses such as the Nrf2 and Sirt3 pathways, triggering extensive lipid peroxidation and severe mitochondrial DNA damage (Angsutararux et al., 2015).

Beyond direct oxidative and nitrative injuries, DOX simultaneously inflicts profound structural abnormalities, including organelle fragmentation, cristae loss, and matrix disruption (Liu and Levine, 2015). This structural decay is accompanied by impaired electron transport chain function at complex I and uncoupled oxidative phosphorylation, triggering a bioenergetic crisis and a precipitous decline in ATP synthesis that directly compromises myocardial contractility (Papadopoulou et al., 1999). Crucially, this pathology is sustained by a comprehensive collapse of the mitochondrial quality control network. For instance, DOX suppresses biogenesis via the PGC-1α/Nrf/Tfam axis (Miragoli et al., 2016; Bonora et al., 2022) and shifts dynamics toward pathological fission through the imbalanced modulation of Drp1/Fis1 and Mfn/Opa1 (Forte et al., 2021). Driven by additional impairments in mitophagy pathways such as PINK1/Parkin and Bnip3/Nix/Fundc1, dysfunctional organelles accumulate rather than clear (Picca et al., 2018; Pfanner et al., 2019). Given that total mitochondrial mass determines DOX tolerance (Xiong et al., 2025), this multifaceted failure severely amplifies cellular vulnerability to toxic insults.

The culmination of excessive reactive oxygen species, calcium overload, and energetic depletion eventually triggers the persistent opening of the mitochondrial permeability transition pore, leading to swelling and the release of cytochrome c to execute apoptosis. Concomitantly, the leakage of damaged mitochondrial DNA synergizes with oxidative stress to activate potent innate immune axes, including the cGAS-STING, NF-κB, and NLRP3 pathways (Shi et al., 2015; Christidi and Brunham, 2021; Luo et al., 2023). Mechanistically, NLRP3 activation drives the canonical caspase-1/gasdermin D (GSDMD) pathway, while apoptotic signaling cross-activates the non-canonical caspase-3/GSDME pathway, collectively triggering cardiomyocyte pyroptosis (Shi et al., 2015). This sterile inflammation is further sustained as dysregulated autophagy-related pathways, such as mTOR and TFEB, crosstalk with NF-κB signaling to lock the cells in a pathogenic oxidative-inflammatory vicious cycle (Christidi and Brunham, 2021). This comprehensive network—intertwining irreversible structural damage, energetic failure, and violent pyroptosis—fundamentally deteriorates the cardiac microenvironment, establishing a critical prerequisite for downstream nuclear epigenetic remodeling events, such as aberrant histone eviction and cleavage.

#### Calcium homeostasis disruption and excitation–contraction uncoupling

Current evidence suggests that the disruption of Ca^2+^ homeostasis in cardiomyocytes is a fundamental molecular and cellular determinant of DIC, significantly contributing to the development of contractile dysfunction (Shinlapawittayatorn et al., 2022) Anthracyclines disrupt Ca^2+^ balance through multiple mechanisms, leading to intracellular Ca^2+^ overload and subsequent impairments in myocardial contraction and relaxation (Pessah, 1992). Specifically, the accumulation of anthracyclines in mitochondria triggers reactive ROS generation, loss of membrane potential, and ATP depletion. This energetic deficit impairs the function of the sarcoplasmic reticulum Ca^2+^-ATPase (SERCA), thereby hindering Ca^2+^ reuptake and elevating free cytoplasmic Ca^2+^ levels (Keung et al., 1991; Hanna et al., 2014). Furthermore, these agents directly modulate ryanodine receptors (RyR) and sodium-calcium exchangers (NCX), leading to aberrant Ca^2+^ leakage from the sarcoplasmic reticulum and disorganized transmembrane Ca^2+^ flux, which together compromise normal cardiac rhythms (Kim et al., 2006). Such Ca^2+^ overload further activates calcium-dependent signaling pathways, including CaMKII, phosphoinositide signaling, and pro-apoptotic cascades, ultimately driving cardiomyocyte apoptosis, necrosis, and even ferroptosis (Villani et al., 1980). As a specific blocker of hyperpolarization-activated cyclic nucleotide-gated (HCN) channels, ivabradine is traditionally utilized for heart rate control; however, emerging evidence highlights its broader cardioprotective potential. Mechanistically, Sripusanapan et al. (2025) demonstrated that ivabradine significantly attenuates DOX-induced sarcoplasmic reticulum (SR) Ca^2+^ leakage and enhances SERCA2A activity by stabilizing RyR2 receptors. This stabilization effectively corrects diastolic Ca2+ overload and improves left ventricular function. Consistent with these mechanistic insights, a recent clinical study has confirmed that ivabradine is a safe and well-tolerated intervention in BC patients receiving DOX therapy who present with tachycardia (Čiburienė et al., 2023). Collectively, these findings underscore the therapeutic potential of targeting calcium regulatory pathways with ivabradine as a promising strategy for mitigating DIC.

#### Dysregulated cell death pathways in DIC

##### Autophagy

In DIC, cardiomyocyte autophagy acts as a double-edged sword. Moderate autophagy maintains cellular homeostasis and mitigates oxidative stress by clearing damaged proteins and organelles. However, excessive or dysfunctional autophagy leads to blocked autophagic flux, triggering ROS accumulation and cardiomyocyte death (Huang et al., 2022; Tan et al., 2025). DOX-induced ROS and oxidative stress can overactivate mitophagy, causing excessive mitochondrial clearance and energy depletion (Luan et al., 2024). DOX impairs the initiation of autophagy by inhibiting the AMPK/mTOR-ULK1 signaling axis, leading to an imbalance characterized by elevated LC3-II levels alongside autophagic flux blockage and p62 accumulation, which ultimately exacerbates myocardial injury. Although Nrf2 activation can partially restore autophagic flux and exert cardioprotective effects, the persistence of lysosomal degradation dysfunction continues to limit the clearance of damaged cellular components and may further promote autophagy-related cell death. As core components of the cytoskeleton, α- and β-tubulin-based microtubules mediate autophagosome transport and lysosomal fusion (Kast and Dominguez, 2017). DOX-induced microtubule disruption severs autophagic flux, resulting in the accumulation of damaged organelles (Larocque et al., 2014). Peng et al. (2024) compared doxorubicin alone and doxorubicin plus low-dose colchicine in hamsters and hiPSC-CMs, and found that low-dose colchicine restored microtubule integrity through dose-dependent depolymerization, enhanced autophagic degradation, and protected against DOX-induced cardiotoxicity, thereby offering a potential therapeutic approach for DOX cardiotoxicity.

##### Apoptosis

Apoptosis is a highly regulated form of programmed cell death characterized by distinct morphological changes, such as chromatin condensation and the formation of apoptotic bodies, which occur without eliciting a significant inflammatory response. In the context of DIC, the intrinsic mitochondrial pathway serves as the predominant driver of cardiomyocyte loss. Following the previously discussed mitochondrial dysfunction and mPTP opening, released cytochrome c assembles with Apaf-1 and pro-caspase-9 to form the apoptosome, which subsequently triggers the caspase-9/3 proteolytic cascade to execute cell death (An et al., 2009). This process is further orchestrated by a sophisticated regulatory network: doxorubicin activates the p53-Bax axis to promote mitochondrial outer membrane permeabilization while simultaneously suppressing GATA4-mediated expression of anti-apoptotic proteins like Bcl-XL (Aries et al., 2004). Combined with the inactivation of the PI3K/Akt pro-survival pathway and the activation of pro-apoptotic JNK/MAPK signaling (Fukazawa, 2003; Fan et al., 2008; Sui et al., 2014), these molecular shifts create an irreversible tilt toward cellular demise, underscoring apoptosis as a primary therapeutic target in mitigating DIC.

##### Necroptosis

Emerging evidence indicates that necroptosis, a regulated form of necrotic cell death, plays a pivotal role in DIC. DOX can activate TNF-α signaling, which, under conditions of inhibited caspase-8 activity, shifts the cell death program toward the RIPK1/RIPK3/MLKL-mediated necroptotic pathway. This transition leads to cardiomyocyte membrane rupture and the amplification of sterile inflammatory responses (Gong et al., 2019). Furthermore, DOX has been shown to directly upregulate RIPK3 activity, which subsequently exacerbates myocardial injury through CaMKII-mediated mitochondrial dysfunction. Together, these processes drive irreversible cardiomyocyte loss, highlighting necroptosis as a fundamental pathological mechanism underlying DOX-related cardiac damage (Zhang et al., 2016).

##### Pyroptosis

Pyroptosis is a form of programmed cell death characterized by inflammasome activation and GSDM-mediated membrane pore formation, leading to the rapid release of pro-inflammatory cytokines such as IL-1βand IL-18 (Shi et al., 2015; Man et al., 2017). In the context of DIC, pyroptosis serves as a critical inflammatory mechanism driven by both canonical and non-canonical pathways. Specifically, DOX has been shown to upregulate the long non-coding RNA TINCR, which recruits the IGF2BP1 protein to facilitate the expression and assembly of the NLRP3 inflammasome. This process subsequently activates caspase-1, leading to the cleavage of GSDMD and the execution of classical inflammatory pyroptosis (Christidi and Brunham, 2021). Beyond the GSDMD-dependent route, DOX can also trigger pyroptosis through a GSDME-dependent mechanism. This occurs via the mitochondrial protein BNIP3, which activates caspase-1, subsequently cleaving GSDME into its pore-forming N-terminal fragment (Christidi and Brunham, 2021). Collectively, these mechanisms position pyroptosis as a critical amplifier of the inflammatory response and a key executioner of cardiomyocyte loss, effectively translating initial cellular stress into sustained myocardial damage in DOX-associated cardiotoxicity.

##### Senescence

DIC is intrinsically linked to irreversible cardiovascular cell senescence, which is primarily driven by DNA damage, telomere dysfunction, and CDKN2A derepression (Jochems et al., 2021). A core mechanism underlying this pathology is mitochondrial damage-induced innate immune activation. During DOX stress, mitochondrial dysfunction—characterized by BAX/BAK-mediated mitochondrial outer membrane permeabilization (MOMP) and the opening of the mPTP—promotes the cytosolic leakage of mtDNA (Bonora et al., 2022; Cosentino et al., 2022; Hom et al., 2011). Cytosolic mtDNA is recognized by cGAS, triggering cGAMP synthesis and STING activation. This initiates the TBK1-IRF3 signaling cascade, upregulating type I interferons and driving the massive secretion of pro-inflammatory cytokines such as IL-6 and IL-8, as well as chemokines such as MCPs and MIPs (Hopfner and Hornung, 2020). This severe mtDNA-cGAS-STING-mediated autoinflammatory “cytokine storm” establishes the senescence-associated secretory phenotype (SASP). Furthermore, it acts as a critical catalyst for premature cardiomyocyte senescence, culminating in progressive microenvironmental deterioration and irreversible cardiac structural remodeling.

##### Ferroptosis

Ferroptosis, a form of iron-dependent programmed cell death characterized by uncontrolled lipid peroxidation, has been increasingly recognized as a pivotal driver of DIC. The central mechanism involves mitochondrial iron overload-driven amplification of lipid peroxidation, precipitated by profound mitochondrial dysfunction. Under oxidative stress, the degradation of the transcriptional repressor Bach1 relieves its inhibitory effect on heme oxygenase-1 (HO-1) transcription, leading to HO-1 upregulation. This enzyme catalyzes heme degradation and releases free ferrous iron (Fe^2+^) upon mitochondrial translocation. This excess Fe^2+^ facilitates the Fenton reaction, heightening reactive ROS generation and triggering lipid peroxidation, thereby establishing the requisite environment for ferroptosis initiation (Tang et al., 2021). Notably, DOX-induced ferroptosis is defined by distinct hallmarks, including a marked increase in lipid peroxidation closely associated with the suppression of glutathione peroxidase 4 (GPX4) activity. As the primary endogenous enzyme for lipid peroxide detoxification, the inactivation of GPX4 prevents the effective clearance of lipophilic radicals, synergizing with excessive ROS accumulation to form a lethal amplification loop (Tadokoro et al., 2020). This process represents a strictly iron-dependent form of cell death, as evidenced by its effective attenuation by iron chelators or the specific ferroptosis inhibitor ferrostatin-1 (Fer-1), confirming the central driving role of iron dyshomeostasis in DIC death (Dixon et al., 2012). Consequently, the ferroptosis pathway, driven by mitochondrial iron imbalance and amplified lipid peroxidation, represents a critical pathological hub in the progression of DIC and offers promising targets for translational therapeutic intervention.

#### DNA damage and epigenetic regulation

Among the chemotherapeutic drugs, anthracyclines are the most commonly associated with cardiotoxicity. On the one hand, they inhibit cardiomyocyte topoisomerase IIβ (Topo IIβ), leading to DNA breaks and disrupting the normal functioning of DNA, on the other hand, they also generate a large amount of ROS, triggering oxidative stress responses, causing lipid peroxidation of the myocardial cell membrane, damaging myocardial Mitochondrial DNA (Stephenson et al., 2024), and DIC is dose-dependent, leading to irreversible heart failure (Cardinale et al., 2015).

Anthracyclines drive cumulative and late-onset cardiotoxicity by inducing profound epigenetic remodeling and chromatin structural collapse. Mechanistically, anthracycline exposure inhibits KDM4C activity, promoting GRHL2-K453 methylation, which recruits Cathepsin L (CTSL) to mediate histone H3 clipping. This structural impairment downregulates glutamate-cysteine ligase (GCL), triggering pathological ROS elevation, and synergizes with Topo IIβ-mediated DNA breaks to facilitate large-scale histone eviction (Li Z. et al., 2025). Concurrently, metabolic reprogramming is not only a hallmark of cancer cells but also directly governs gene regulation through epigenetic mechanisms. A key example is lactate, which was once considered merely a glycolytic byproduct but is now recognized as an important signaling metabolite. In TNBC, enhanced glycolytic flux and LDH-A upregulation are closely linked to poor prognosis and significantly elevated levels of histone lactylation. This lactate-derived modification synergizes with active epigenetic markers, such as H3K27ac, to promote chromatin openness and drive oncogenic transcription, thereby accelerating tumor progression (Li et al., 2026). A critical determinant of cardiac vulnerability lies in the failure of histone homeostasis regulation. Unlike chemoresistant tumor cells, which leverage the NASP-INO80-PARP1 axis for efficient histone turnover, cardiomyocytes exhibit an inherent deficiency in the NASP-mediated supply pathway. Consequently, cardiomyocytes are unable to compensate for the physical histone depletion caused by Topo IIβ-mediated eviction and CTSL-mediated clipping. Furthermore, this regulatory failure renders heart cells incapable of rectifying the epigenetic “misreading” triggered by aberrant histone lactylation. The convergence of these defects ultimately leads to the collapse of chromatin structural integrity and irreversible transcriptional instability, underpinning the molecular pathology of DIC (Moser et al., 2025).

### Targeted therapy

Targeted therapy–associated cardiotoxicity, particularly during trastuzumab treatment, involves coordinated interactions among cardiomyocytes, cardiac macrophages, vascular endothelial cells, and cardiac fibroblasts. HER2 blockade disrupts PI3K/AKT-dependent survival signaling in cardiomyocytes, promoting cellular stress and the release of damage-associated molecular patterns (DAMPs), which activate resident macrophages—especially the CD74^+^ subset enriched in IFN-γ responsive genes. Engagement of the CD74-associated signaling axis enhances JAK-mediated STAT1 phosphorylation and nuclear translocation, driving transcription of pro-inflammatory mediators in cooperation with NF-κB (Zhu M. et al., 2025). In parallel, trastuzumab-exposed endothelial cells upregulate pentraxin 3 (PTX3), an acute-phase mediator that facilitates leukocyte recruitment and reinforces innate immune activation. Elevated PTX3 may further potentiate STAT1-dependent inflammatory signaling in macrophages, amplifying cytokine production and sustaining myocardial inflammation (Xu et al., 2023). Macrophage-derived cytokines subsequently promote cardiac fibroblast activation and extracellular matrix accumulation, leading to increased myocardial stiffness, while persistent cardiomyocyte stress drives pathological remodeling and cardiac functional decline.

This immune–vascular–myocardial framework extends beyond trastuzumab. The expanding spectrum of breast cancer targeted therapies—including HER2-directed antibody–drug conjugates (ADCs), Cyclin-dependent kinases 4 and 6 (CDK4/6) inhibitors, Poly (ADP-ribose) polymerase (PARP) inhibitors, and phosphatidylinositol 3-kinase/mechanistic target of rapamycin (PI3K/mTOR) inhibitors—may perturb distinct components of this cellular network, thereby influencing myocardial immune homeostasis and remodeling susceptibility.

#### Anti-HER2 agents

The pathogenesis of trastuzumab-induced cardiotoxicity is a complex and multifaceted process involving several interrelated mechanisms. Under physiological conditions, HER2 signaling plays a critical role in maintaining cardiomyocyte survival, contractile function, and adaptive responses to stress. This protective signaling is primarily mediated by the formation of HER2/HER4 heterodimers—triggered by the binding of neuregulin-1 (NRG1) to HER4—as well as HER2/HER2 homodimers, which activate the PI3K/AKT/mTOR and RAS/RAF/MEK/MAPK pathways (Pamungkas et al., 2025). However, treatment with HER2-targeted monoclonal antibodies, such as trastuzumab and pertuzumab, disrupts these protective networks. Trastuzumab binds to the extracellular domain IV of HER2, exerting steric hindrance that blocks both homodimerization and NRG1-induced HER2/HER4 heterodimerization (Moasser, 2007). As a result, downstream AKT, mTORC1, and MAPK signaling is markedly suppressed, shifting cardiomyocytes toward a pro-apoptotic state characterized by myofibrillar loss and adverse structural remodeling. In parallel, trastuzumab induces mitochondrial dysfunction and promotes excessive oxidative stress. Inhibition of AKT signaling weakens the Nrf2–HO-1 antioxidant axis (Yang et al., 2020), lleading to impaired mitochondrial redox balance and the accumulation of ROS. Elevated oxidative stress is further reinforced by increased Ang II levels, which activate NADPH oxidase and amplify ROS production. This oxidative burden is closely linked to iron dysregulation, lipid peroxidation, and the upregulation of prostaglandin-endoperoxide synthase 2 (PTGS2) (Liu Y. et al., 2024). Beyond direct cardiomyocyte injury, trastuzumab also affects the cardiac microvasculature. Blockade of HER2 signaling in endothelial cells triggers the paracrine release of PTX3, which acts on neighboring cardiomyocytes to reduce intracellular calcium levels and impair contractilitya (Wills et al., 2021).

#### ADCs

ADCs represent a paradigm shift in precision oncology, leveraging the specificity of monoclonal antibodies to deliver potent cytotoxic payloads. While designed to spare healthy tissues, emerging clinical evidence highlights a growing concern regarding their cardiovascular safety. The pathogenesis of ADCs-induced cardiotoxicity is multifactorial, encompassing target-dependent, payload-driven, and off-target mechanisms (Nguyen et al., 2023). Although some toxicities arise from on-target binding to antigens expressed basally on cardiomyocytes, the vast majority of dose-limiting toxicities stem from non-specific off-target effects. Remarkably, over 99% of administered ADCs undergo non-specific endocytosis and metabolism in normal tissues rather than tumor-specific uptake. This off-target sequestration is primarily driven by the physicochemical properties of the ADCs; a high DAR significantly increases molecular hydrophobicity and surface charge, thereby accelerating non-specific cellular uptake and subsequent payload accumulation within the cardiac microenvironment. Molecular sequestration is further modulated by specific receptor-mediated pathways independent of the target antigen. The interaction between the antibody’s Fc domain and Fc-γ receptors on resident cardiac macrophages or megakaryocytes mediates localized payload release, while Mannose Receptor mediated endocytosis in sinusoidal endothelial cells serves as another critical driver of systemic tissue insult. These processes are intimately linked to linker stability; while cleavable linkers exploit the bystander effect to overcome tumor heterogeneity, insufficient stability leads to premature systemic payload leakage, narrowing the therapeutic window and precipitating direct cardiomyocyte injury (Long et al., 2024).

Beyond direct cytotoxicity, ADCs function as potent immunomodulators by triggering ICD and the release of DAMPs like calreticulin and HMGB1. This process facilitates anti-tumor immunity by activating dendritic cells (DCs) via the GEF-H1/RhoA signaling pathway, yet it concurrently risks inducing localized inflammatory surges and cytokine release within the myocardium. Finally, a patient’s prior therapeutic history—including cumulative damage from anthracyclines or radiotherapy—severely depletes cardiac reserve, synergistically increasing myocardial susceptibility to subsequent ADC-induced insult.

#### CDK4/6 inhibitors

CDK4/6 are critical regulators of cell cycle progression, driving the transition from the G1 to the S phase, and their dysregulation is a central driver in oncogenesis. Targeting this pathway with CDK4/6 inhibitors has significantly transformed the management and natural history of HR+/HER2- BC; however, prolonged patient survival has increasingly unmasked potential cardiac adverse effects. Multiple meta-analyses have demonstrated that the administration of CDK4/6 inhibitors is associated with an increased risk of developing arrhythmias (Murad et al., 2024; Hoo et al., 2026). Among this drug class, ribociclib is predominantly associated with cardiovascular toxicity manifesting as QT interval prolongation (Dent et al., 2025). The pathogenesis of this specific proarrhythmic effect is multifaceted, involving both transcriptional dysregulation and direct ion channel interference. On a genetic level, ribociclib induces the aberrant expression of key genes associated with long QT syndrome. Microarray analyses have demonstrated that ribociclib downregulates KCNH2, the gene encoding the hERG potassium channel, while concurrently upregulating SCN5A, which encodes the cardiac Nav1.5 sodium channel, and SNTA1, a key regulator of sodium channel complex stability (Tao et al., 2017). Furthermore, drug-induced QT prolongation is frequently mediated by the direct blockade of potassium channels encoded by hERG (Gintant et al., 2006). Comparative safety profiling has explicitly shown that ribociclib exerts a distinct inhibitory effect on the hERG channel (Santoni et al., 2019). Together, these transcriptomic alterations and direct disruptions of potassium and sodium channel dynamics delay ventricular repolarization, providing a comprehensive mechanistic explanation for ribociclib-induced electrophysiological instability.

#### PARP inhibitors and PI3K/mTOR inhibitors

The clinical application of PARP inhibitors and PI3K/mTOR inhibitors in oncology is precisely tailored to specific molecular subtypes, yet both classes present unique vascular and metabolic risks. PARP inhibitors are primarily indicated for HER2- (especially triple-negative) BC patients harboring germline or somatic BRCA mutations, while also serving as a cornerstone for maintenance therapy in ovarian cancer (Wang F. et al., 2025). Their cardiovascular profile is characterized by systemic hypertension, driven by the impairment of DNA-repair pathways within the vascular endothelium. This disruption precipitates localized oxidative stress and compromises endothelial integrity, leading to increased peripheral resistance and arterial stiffness.

In contrast, PI3K/mTOR inhibitors are utilized in HR+ and HER2-advanced BC to counteract dysregulated signaling. However, since the PI3K/AKT/mTOR axis is the central regulator of systemic nutrient homeostasis, its pharmacological blockade induces profound metabolic and bioenergetic disturbances (Dey et al., 2017). Specifically, PI3K inhibition suppresses the translocation of (glucose transporter type 4) GLUT4 to the cell membrane in skeletal muscle and adipose tissue, leading to acute insulin resistance and hyperglycemia (Yang et al., 2024). Concurrently, the inhibition of mTOR interferes with hepatic fatty acid metabolism and lipid sensing, resulting in hyperlipidemia.

Beyond these systemic shifts, these agents trigger bioenergetic and autophagy failure within cardiomyocytes and subsequent apoptosis. Furthermore, the disruption of mitochondrial ATP production exacerbates cellular energy deficits (Morita et al., 2017), creating an energy-deficient environment that increases long-term susceptibility to ischemic stress and contractile dysfunction.

### Endocrine therapy

Endocrine therapy represents a cornerstone of long-term treatment for hormone receptor–positive BC and mainly includes selective estrogen receptor modulators (SERMs), aromatase inhibitors (AIs), gonadotropin-releasing hormone (GnRH) agonists, and selective estrogen receptor degraders (SERDs). Although these strategies demonstrate distinct antitumor efficacy, their cardiovascular effects differ substantially and extend beyond simple estrogen deprivation.

Endocrine therapy reshapes the systemic endocrine–immune milieu involving cardiomyocytes, fibroblasts, and macrophages. In the tumor microenvironment, infiltrating CD68^+^ macrophages enhance immunoreactive aromatase (irARO) expression, increasing local estrogen production and sustaining inflammatory signaling (Mor et al., 1998). At the same time, therapy-associated metabolic adverse effects—particularly insulin resistance, visceral adiposity, and weight gain—may promote secondary obesity and chronic low-grade inflammation. Excess adiposity facilitates myofibroblast differentiation through activation of the TGF-β1/SMAD3/miR-140 axis, reinforcing fibrotic remodeling (Avagliano et al., 2020). Systemically, estrogen deprivation and metabolic dysregulation impair cardiomyocyte function, while macrophage-derived cytokines stimulate cardiac fibroblast activation and extracellular matrix deposition. Thus, endocrine therapy–related cardiac dysfunction can be understood within a multicellular framework characterized by dynamic crosstalk among cardiomyocytes, fibroblasts, and immune cells under hormonal and metabolic stress.

#### SERMs

SERMs, represented by tamoxifen, have been shown in multiple studies to improve lipid profiles by reducing low-density lipoprotein cholesterol levels and carotid intima–media thickness, thereby exerting a potential protective effect against atherosclerosis (Clarke et al., 2001; Sahebkar et al., 2017; Ruhstaller et al., 2019). However, the procoagulant properties of tamoxifen may increase the risk of VTE and ischemic stroke (Deitcher and Gomes, 2004), conferring a “double-edged sword” characteristic to its cardiovascular outcomes. Compared with AIs, tamoxifen is associated with a significantly higher risk of VTE (Deitcher and Gomes, 2004; Xu et al., 2019). Moreover, evidence regarding the cardiovascular safety of tamoxifen remains conflicting. Previous studies have reported an association between tamoxifen treatment and QT interval prolongation (Slovacek et al., 2008; Grouthier et al., 2018). Mechanistic investigations indicate that tamoxifen and its metabolites can bind to intracellular domains of voltage-gated sodium (Nav) channels, selectively inhibiting sodium currents, thereby slowing the upstroke of the cardiac action potential and markedly prolonging the cardiac excitation cycle in a dose-dependent manner. These electrophysiological alterations suggest impaired atrioventricular nodal and ventricular conduction, potentially increasing the risk of conduction block and arrhythmias (Xie et al., 2023).

At the molecular level, emerging evidence highlights a context-dependent regulatory role of the PI3Kα signaling pathway in tamoxifen-associated cardiac effects. Experimental studies have demonstrated that PI3Kα deficiency leads to severe cardiac dysfunction, whereas PI3Kα mediates dose-sensitive repair mechanisms and interacts with ERK1/2 and AMPK signaling pathways to exert cardioprotective effects under stress conditions. Low-dose tamoxifen (40 mg/kg) exerts minimal adverse effects on systolic and diastolic function, with its potential protective role largely dependent on ERK1/2 activation, which promotes cardiomyocyte survival and compensatory adaptation. In contrast, high-dose tamoxifen exposure (60 mg/kg) abolishes ERK1/2-mediated compensation, results in sustained inhibition of Akt activity, and provides insufficient AMPK compensation. Therefore, therapeutic strategies targeting PI3Kα or ERK1/2 signaling may represent potential approaches to mitigate tamoxifen-related cardiotoxicity (McLean et al., 2015).

#### AIs

Als exert antitumor effects by markedly suppressing peripheral estrogen synthesis and constitute the primary endocrine therapy for postmenopausal BC patients. In animal models, letrozole treatment induces increased lipid deposition and disorganized myocardial fiber arrangement in cardiac tissue, accompanied by upregulation of cardiac hypertrophy markers and fibrosis-related genes, suggesting that letrozole may act as a key driver of cardiac structural remodeling (Mukherjee et al., 2022). In addition, aromatase inhibitor–associated musculoskeletal symptoms (AIMSS) have been widely reported and are primarily attributed to estrogen deficiency–induced disruption of bone–joint homeostasis and enhanced inflammatory responses. By suppressing estrogen production, AIs promote osteoclast activation, impair osteoblast function, and accelerate articular cartilage degeneration, thereby increasing the risk of musculoskeletal pain (Zhu et al., 2020). Under hypoestrogenic conditions, proinflammatory cytokines such as TNF-α and IL-1β are upregulated, while imaging studies often reveal tendon thickening and joint effusion, indicating the involvement of localized inflammatory processes in pain development (Reinbolt et al., 2018). Furthermore, genetic susceptibility may contribute to Aromatase Inhibitor-Induced Musculoskeletal Symptoms (AIMSS), as variants in genes such as BLS1 and TRPV3 have been associated with AI-related arthralgia, potentially through amplification of synovial inflammation via the NF-κB signaling pathway.

#### GnRH agonists

Ovarian function suppression is primarily used in premenopausal BC patients and is often combined with SERMs or AIs to induce a state of “pharmacological menopause” and substantially reduce circulating estrogen levels. An observational study in prostate cancer patients receiving GnRH agonists or antagonists reported a significantly higher incidence of cardiovascular events in the agonist group compared with the antagonist group. Patients aged 65–85 years constituted the majority of cardiovascular events, with particularly high proportions of ischemic stroke and myocardial infarction. Elderly patients (>65 years) or those with pre-existing CVD should therefore be cautious when treated with GnRH agonists (Cicione et al., 2023). Notably, these conclusions are primarily derived from prostate cancer populations, and the cardiovascular risk associated with OFS in BC patients requires further investigation and validation.

#### SERDs

SERDs, such as fulvestrant, bind to estrogen receptors (ERs), induce conformational changes, and promote receptor degradation. Basu et al. demonstrated in wild-type zebrafish that SERDs suppress nuclear ERα-dependent transcriptional regulation in cardiomyocytes, leading to direct downregulation of key pacemaker genes, including hcn4 and tbx3, ultimately resulting in bradycardia (Basu et al., 2026). These findings suggest that SERD-related cardiac risks warrant particular attention, especially in patients with coexisting traditional cardiovascular risk factors.

### Immunotherapy

In recent years, with the increasing use of immune checkpoint inhibitors (ICIs), such as PD-1/PD-L1 inhibitors, in the treatment of TNBC, their unique immune-related cardiovascular toxicities have emerged as a new potential risk factor for CVD in BC patients. Particular attention should be paid to ICI-associated myocarditis (ICIAM), ICIAM results from multi-level immune dysregulation, with its core mechanisms encompassing cardiac antigen–driven T cell–macrophage immune interactions, humoral immunity and antigen cross-reactivity–mediated myocardial injury, as well as the involvement of inflammatory mediators, proinflammatory signaling pathways, and oxidative stress. Early recognition of symptoms and timely initiation of corticosteroid therapy are key to improving prognosis (Moslehi et al., 2021; Cozma et al., 2022; Lei et al., 2024).

Multi-omics analyses have revealed that autoreactive CD8^+^ T cells, antigen-presenting cells (APCs; such as dendritic cells), and cardiac fibroblasts together constitute a central immunological hub underlying immune therapy–related cardiovascular toxicity and myocardial injury (Tong et al., 2025). Increasing evidence indicates that immune crosstalk between T lymphocytes and macrophages represents a key driving force in both infectious and autoimmune diseases. In ICIAM, macrophages function as professional antigen-presenting cells, delivering co-stimulatory signals (e.g., B7–CD28) to T cells via major histocompatibility complex (MHC) molecules, thereby sustaining aberrant T-cell activation (Yao et al., 2014). Activated T cells secrete high levels of proinflammatory cytokines, including IFN-γ and TNF-α, which further activate macrophages and induce the release of chemokines such as CXCL9 and CXCL10, as well as inflammatory mediators including IL-6 and IL-12. This reciprocal interaction forms a positive feedback loop that amplifies immune cell recruitment and myocardial inflammation (Sreejit et al., 2022; Frisancho-Kiss et al., 2006). Clinically, significantly elevated levels of CXCL9 and CXCL10 have been detected in both cardiac tissue and peripheral blood of patients with ICIAM, promoting sustained infiltration of T cells and monocytes into the myocardium via the CXCR3 receptor. Although this chemokine axis normally contributes to antitumor immunity within the tumor microenvironment, its dysregulated activation in the context of ICI therapy may result in off-target immune attacks against the heart, thereby aggravating immune-mediated myocardial injury (Kao et al., 2010; Abou Shousha et al., 2023). Moreover, infiltrating macrophages maintain a proinflammatory microenvironment and, in later disease stages, promote cardiac fibroblasts to upregulate angiopoietin-like protein 2 (ANGPTL2) and activate NF-κB signaling. This enhances chemokine secretion, facilitates further immune cell recruitment, and drives myocardial interstitial fibrosis, ultimately leading to progressive cardiac dysfunction (Horiguchi et al., 2023).

At the level of humoral immunity, ICI therapy disrupts B-cell immune tolerance and induces the production of anti-cardiac autoantibodies. In PD-1–deficient mouse models, the emergence of anti–cardiac troponin I (cTnI) antibodies is directly associated with the development of dilated cardiomyopathy. These autoantibodies target cardiomyocyte structural proteins, including cTnI and cardiac myosin, leading to contractile dysfunction and structural remodeling through disruption of calcium homeostasis and persistent activation of the cAMP/PKA signaling pathway (Okazaki et al., 2003). In addition, the Fc domains of autoantibodies can be recognized by Fc receptors expressed on macrophages and NK cells, mediating antibody-dependent cellular cytotoxicity (ADCC) and reactive ROS generation, which ultimately result in cardiomyocyte apoptosis or necrosis (Sun et al., 2021). Antigen–antibody immune complexes may also activate the complement cascade, leading to direct cardiomyocyte injury via membrane attack complex (MAC) formation or indirect amplification of inflammation through recruitment of neutrophils. Notably, PD-1 blockade has been shown to enhance T-cell–mediated ADCC, further exacerbating antibody-driven myocardial injury (Michel et al., 2022).

From the perspective of cross-reactive immunity, homologous antigen epitopes shared between tumor tissue and myocardium can be recognized by identical T cell receptors (TCRs), enabling activated T cells to simultaneously target tumor-associated and cardiac antigens, thereby triggering off-target immune-mediated myocardial injury (Blum et al., 2024). In immune checkpoint ICIAM, shared antigens are considered a key driver of aberrant cardiac immune responses. Cancer immunotherapy, particularly immune checkpoint blockade, leads to robust activation of tumor-specific T cells while concurrently promoting the release of cardiac self-antigens, such as α-myosin heavy chain (α-MyHC) (Tong et al., 2025). Owing to structural homology or epitope similarity between certain tumor antigens and cardiac proteins, these activated T cells may misrecognize cardiomyocytes during antitumor immune responses, resulting in unintended immune attacks on the heart. Following blockade of PD-1 and/or CTLA-4, CD8^+^ cytotoxic T cells lose immune restraint and become excessively activated. These cells directly induce cardiomyocyte apoptosis through the perforin–granzyme B and Fas–FasL pathways, while simultaneously secreting proinflammatory mediators such as IFN-γ, FasL, and CD40L, which activate downstream signaling cascades including STAT1 and NF-κB. This inflammatory amplification not only exacerbates cardiomyocyte death but also promotes further release of cardiac antigens, thereby sustaining T cell activation and establishing a self-perpetuating cycle of immune dysregulation and myocardial damage (Wong et al., 2023; Zhang Z. et al., 2025; Ma et al., 2024).

### Radiation therapy

Radiotherapy plays an indispensable role in BC treatment, particularly as a crucial adjuvant therapy following breast-conserving surgery. However, radiotherapy-induced cardiovascular toxicity remains a significant clinical challenge, primarily mediated by severe deoxyribonucleic acid damage, programmed cell death, and the aberrant activation of pervasive inflammatory and fibrotic networks. Mechanistically, the ionizing radiation generated during treatment directly interacts with cellular DNA or indirectly induces double-strand breaks through free radical-mediated pathways, leading to a massive accumulation of genetic damage, intense oxidative stress, and focal ischemia. These initial structural insults critically disrupt the functional integrity of cardiomyocytes, ultimately executing the apoptotic program (Thoms and Bristow, 2010; Saiki et al., 2017; Yeboa and Evans, 2016). Besides apoptosis, radiation can induce necroptosis in breast cancer cells. MLKL functions as the key executor of necroptosis and mediates the crosstalk between apoptotic and necroptotic signals, which determines the killing effect of radiotherapy and makes MLKL a potential target against radioresistance (El Feky et al., 2024). Concurrently, this radiation exposure provokes the robust expression of a vast array of cytokines and growth factors within the myocardial tissue, notably TNF-α, type I IFNs, TGF-β, GM-CSF, and a broad spectrum of interleukins ranging from IL-1 to IL-18 (Di Maggio et al., 2015). This hyperactive immune response synergistically exacerbates the collateral tissue injury by further amplifying deoxyribonucleic acid strand breaks and perpetuating the continuous elevation of reactive oxygen species (Zhou L. et al., 2024). Furthermore, these radiation-induced signaling molecules potently stimulate the recruitment and hyperactivation of diverse circulating immune populations, primarily macrophages, neutrophils, and lymphocytes, establishing a relentless cycle of chronic sterile inflammation that severely impairs long-term functional integrity (Verginadis et al., 2025).

Importantly, endothelial cells are highly radiosensitive and develop impaired nitric oxide production and junctional disruption. Endothelial dysfunction not only compromises microvascular perfusion but also disrupts endothelial–cardiomyocyte metabolic coupling, thereby linking vascular injury to secondary cardiomyocyte energetic stress. At the vascular level, ionizing radiation vigorously suppresses nitric oxide production within vascular endothelial cells while exacerbating oxidative stress, culminating in profoundly impaired endothelium-dependent vasodilation (Azimzadeh et al., 2015). Simultaneously, the downregulation of critical intercellular junction proteins, such as vascular endothelial cadherin, dramatically increases vascular permeability and dismantles the endothelial barrier function. This radiation-induced endothelial injury promotes the upregulation of adhesion molecules including ICAM-1 and VCAM-1, facilitating monocyte adhesion and transmigration and thereby strengthening endothelial–immune cell interactions within the irradiated myocardium. Persistent immune cell infiltration triggers macrophage activation and accelerates lipid deposition, contributing to atherosclerotic plaque formation and progressive luminal narrowing within the coronary arteries (Stefan et al., 2023; Basavaraju and Easterly, 2002). Compounding this vascular toxicity, radiotherapy profoundly disrupts lymphatic homeostasis by inhibiting the essential lymphangiogenesis mediated by VEGFR-3 and its ligand VEGF-C (Kishimoto et al., 2018; Narayanan et al., 2019). The dysregulation of the PAI-1/ID-1 axis further hyperactivates the Notch signaling pathway, coercing lymphatic endothelial cells into premature senescence and functional failure (Scharpfenecker et al., 2009). Together, these microvascular and lymphatic deteriorations severely restrict interstitial fluid clearance, thereby aggravating local myocardial inflammation and edema.

In parallel, macrophage-derived TGF-β and PDGF drive fibroblast-to-myofibroblast differentiation. Activated fibroblasts deposit extracellular matrix proteins and secrete additional profibrotic mediators, including bFGF and CTGF, which act in a paracrine manner on adjacent cardiomyocytes and impair excitation–contraction coupling. Through this macrophage–fibroblast–cardiomyocyte signaling network, sustained inflammatory stimulation progressively translates into structural remodeling of the myocardium. Following radiation exposure, infiltrating immune cells together with stressed resident cardiac cells release a broad spectrum of profibrotic mediators, including TGF-β, PDGF, bFGF, and CTGF, further reinforcing fibroblast activation and proliferation. The resulting excessive extracellular matrix deposition increases myocardial stiffness and contributes to the clinical manifestations of radiation-induced myocarditis and pericarditis (Stewart et al., 2010; Yarnold and Vozenin Brotons, 2010; Massagué and Sheppard, 2023; Martin et al., 2000). Over time, this unresolved fibrotic cascade accelerates adverse cardiac remodeling and irreversible functional decline. Beyond these severe structural and cellular injuries, emerging evidence underscores that thoracic radiotherapy simultaneously disrupts intrinsic cardiac autonomic regulation. Comprehensive assessments evaluating deceleration capacity, acceleration capacity, and overall heart rate variability have revealed a definitive radiation-induced suppression of parasympathetic activity coupled with a pathological enhancement of sympathetic tone (Wu et al., 2023). This profound cardiovascular autonomic dysfunction is likely driven by both direct radiation injury to the vagus nerve terminals and the pervasive systemic elevation of neurotoxic inflammatory cytokines, particularly TNF-α and IL-6, which collectively dismantle the delicate balance of the autonomic nervous system.

### Effects of combined therapies

In the standardized treatment of BC, combination therapies have become a cornerstone for improving therapeutic efficacy. However, compared with monotherapy, combination treatments exhibit unique synergistic cardiotoxic characteristics, including the accumulation of multi-pathway damage, complex patterns of cell death, and impaired myocardial repair capacity. Chemotherapy combined with targeted therapy can markedly exacerbate cardiac dysfunction, displaying a “direct injury plus repair inhibition” synergistic effect that accelerates left ventricular functional decline and limits recovery.

Recent translational evidence has shed light on the molecular underpinnings of these clinical observations. Specifically, a study by Szczepaniak et al. (2022) revealed a novel mechanism of vascular toxicity induced by neoadjuvant chemotherapy (NACT). Their research found that docetaxel-based combination regimens trigger the activation of the NOX4 pathway, significantly increasing the production of vascular reactive ROS. This oxidative stress environment promotes the inhibitory phosphorylation of eNOS at the Thr495 site, leading to eNOS dysfunction and reduced NO bioavailability. The resulting imbalance of the NOX4-ROS-eNOS axis provides a mechanistic explanation for the impaired endothelium-dependent vasodilation and chronic hypertension often observed in these patients.

These vascular insights further clarify why cardiotoxicity from chemotherapy combined with radiotherapy primarily manifests as a “time-space cumulative effect,” where subclinical myocardial injury and microvascular damage interact to accelerate chronic heart failure. Similarly, endocrine therapy combined with CDK4/6 inhibitors or sequential chemotherapy is associated with cumulative metabolic-related risks; here, estrogen suppression and vascular smooth muscle dysfunction act synergistically with the aforementioned NOX4-mediated oxidative stress to aggravate atherosclerosis and long-term left ventricular dysfunction. Furthermore, chemotherapy combined with immunotherapy or PARP inhibitors highlights an inflammation-amplifying effect, where endothelial injury and ion channel disturbances increase the risk of arrhythmias and thrombotic events.

Overall, combination therapy–related cardiotoxicity represents a systemic, cumulative, and multi-phenotypic effect encompassing acute injury, chronic risk accumulation, and the impairment of myocardial self-repair. These findings, particularly the identification of specific signaling axes like NOX4-ROS-eNOS, underscore the need for clinicians to monitor synergistic cardiotoxic effects and prioritize targeted prevention when designing combination treatment regimens.

## Diagnosis

### Biomarkers

In patients with BC complicated by CVD, the early recognition of cancer treatment–related cardiac dysfunction (CTRCD) is of critical clinical importance. Compared with women without BC, women with BC have a higher risk of developing heart failure and/or cardiomyopathy, stroke, arrhythmias, cardiac arrest, VTE, as well as all-cause mortality. Importantly, these risks vary according to a history of chemotherapy, radiotherapy, or endocrine therapy (Greenlee et al., 2022). The 2022 ESC Cardio-Oncology Guidelines recommend risk stratification before therapy initiation, with CTRCD defined as either a ≥10% decline in left ventricular ejection fraction (LVEF) to <53% or a >15% relative reduction in global longitudinal strain (GLS) (Lyon et al., 2022). Cardiac biomarkers remain central to early detection. cTn is the gold standard for the diagnosis of myocardial injury. Elevated cTn levels are associated with systolic dysfunction in patients undergoing anticancer therapy and carry important value for predicting left ventricular dysfunction (Rakisheva et al., 2025). In patients treated with anthracycline chemotherapy or trastuzumab targeted therapy, cTn exhibits early and specific elevation, and its dynamic changes can effectively the risk of cardiovascular toxicity in BC patients with CVD (Xia et al., 2025). NT-proBNP sensitively reflects myocardial remodeling and cardiac dysfunction, serving as a reliable indicator for monitoring the progression of chronic heart failure after combined chemotherapy and radiotherapy. A prospective study further demonstrated that NT-proBNP is a reliable predictor of CTRCD inpatients receiving anthracyclines or trastuzumab, with each doubling of baseline NT-proBNP associated with a 56% higher risk of CTRCD (Demissei et al., 2020). However, elevated natriuretic peptide levels in cancer patients can be caused by multiple cardiac and non-cardiac factors, which may reduce the diagnostic accuracy of natriuretic peptides for CTR-CVT (Mueller et al., 2019). In addition to traditional biomarkers such as NT-proBNP and cardiac troponins, soluble growth stimulation expressed gene 2 protein (ST2), growth differentiation factor-15 (GDF-15), myeloperoxidase (MPO), soluble fms-like tyrosine kinase-1 (sFlt-1), placental growth factor (PlGF), galectin-3, arginine–nitric oxide metabolites, heart-type fatty acid–binding protein (H-FABP), glycogen phosphorylase isoenzyme BB (GPBB), and microRNAs may also serve as novel candidate biomarkers reflecting CTR-CVT.

#### sST2

As a member of the IL-1 receptor family, sST2 is a specific biomarker reflecting myocardial mechanical stress and fibrosis. By regulating the IL-33/ST2 signaling pathway, sST2 participates in the crosstalk between cardiovascular injury and tumor-associated inflammation. Numerous studies have demonstrated that sST2 is not only closely associated with short-term adverse prognosis in patients with heart failure, but also an independent risk factor for predicting major adverse cardiovascular events (MACE) (Acharya et al., 2025; Ohnewein et al., 2025). In BC patients receiving antitumor therapy such as anthracyclines and trastuzumab, sST2 can sensitively reflect chemotherapy-induced myocardial injury, and shows potential value, especially in the risk assessment of early cardiovascular complications including heart failure (Frères et al., 2018; Chen C. et al., 2023).

#### GDF-15

GDF-15 is a member of the TGF-β superfamily. It is highly expressed in various solid tumors and the placenta, and is involved in both the regulation of tumor proliferation and myocardial ischemic stress response. GDF-15 is not only highly expressed in the tumor microenvironment but also inhibits the activity and migration of T cells through multiple mechanisms, thereby promoting tumor immune evasion (Melero et al., 2025). Several studies in BC patients treated with trastuzumab, doxorubicin, or the combination of trastuzumab and pertuzumab have suggested that GDF-15 may serve as a candidate biomarker for predicting an elevated risk of antitumor therapy-related cardiotoxicity (Ky et al., 2014; Putt et al., 2015; Kirkham et al., 2022; Tian C. et al., 2024). Nevertheless, current evidence remains limited and requires further substantiation and validation; larger-scale clinical studies are warranted to draw definitive conclusions.

#### MPO

MPO, an inflammatory enzyme released following neutrophil activation, induces oxidative stress-mediated vascular endothelial injury, accelerates atherosclerosis, and promotes the proliferation and invasion of BC cells (Nettersheim et al., 2023). Gustafson et al. profiled 92 cardiovascular-related proteins in patients with early-stage HER2-positive BC receiving anthracycline-trastuzumab combination therapy. Utilizing an artificial intelligence (AI)-driven algorithm, they identified a three-biomarker panel comprising MPO, ANGPT2 (angiopoietin-2), and ENG (soluble CD105/endoglin) that predicted CTRCD with an area under the receiver operating characteristic curve (AUROC) of 0.972 (95% confidence interval: 0.948–0.991) (Gustafson et al., 2025). However, this study was constrained by population and geographic limitations. Furthermore, it merely established an association between these biomarkers and CTRCD, without elaborating on the specific mechanisms through which ANGPT2, MPO, and ENG mediate CTRCD. Additional investigations are warranted to validate their potential as therapeutic targets.

#### sFlt-1 and PIGF

sFlt-1 is a spliced variant of VEGFR-1/FLT-1 that lacks transmembrane and intracellular domains. Acting as a decoy receptor, sFlt-1 neutralizes the biological activities of PlGF and VEGF. By binding to PlGF and VEGF, sFlt-1 blocks their interaction with membrane-bound receptors FLT-1/FLK-1, thereby inhibiting pathological angiogenesis associated with tumors and atherosclerosis (Luttun et al., 2002). In contrast, PlGF promotes angiogenesis in ischemic tissues by activating the FLT-1 signaling pathway and induces macrophages to secrete pro-angiogenic factors such as TNF-α and MCP-1 (De Falco, 2012; Dewerchin and Carmeliet, 2014). Within the tumor microenvironment, PlGF can be released by injured endothelial cells. It enhances FLK-1-mediated angiogenic signaling by competitively displacing VEGF and participates in regulating the repair of endothelial and smooth muscle cells in response to ischemic injury (Tudisco et al., 2014). In pancreatic cancer, PlGF accelerates disease progression by promoting perineural invasion (PNI), particularly extratumoral perineural invasion (ET-PNI) and intraneural invasion (INI). High PlGF expression is significantly associated with poor prognosis and serves as an independent predictor of disease-free survival (DFS) in patients with resectable pancreatic cancer (Göhrig et al., 2024). However, the precise role and molecular mechanisms of PlGF in BC remain to be further elucidated.

#### Galectin-3

Galectin-3, a key member of the β-galactoside-binding lectin family, accelerates atherosclerotic plaque progression by promoting macrophage infiltration, foam cell formation, and vascular inflammation. Meanwhile, it suppresses T cells function and mediates immune evasion in the tumor microenvironment (Wang X. et al., 2025). Xu et al. (2024) established a leukemia inhibitory factor/galectin-3 double-knockout BC model and found that Galectin-3 deficiency reduced neural activity in brain regions including the paraventricular nucleus (PVN) and nucleus tractus solitarius (NTS), thereby delaying BC progression. Although Galectin-3 has been validated as a reliable predictive biomarker in CVD such as heart failure, sufficient evidence is still lacking to support its role as a specific biomarker for DIC in BC patients (Patel et al., 2021; Wu et al., 2022). Further studies combining other biomarkers or extending the follow-up period are warranted to validate its clinical utility.

#### Arginine–nitric oxide metabolites

Arginine is involved in pathways including the urea cycle, NO synthesis, and polyamine biosynthesis, regulating ammonia metabolism, signal transduction, and cell proliferation (Seyfried et al., 2014). Altered levels of key metabolites in the L-arginine-NO pathway are associated with tumor progression or therapeutic response in breast, ovarian, and gastric cancers (Bednarz-Misa et al., 2021; Chen L. et al., 2023). Studies have shown that arginine secreted by cancer cells is metabolized by tumor-associated macrophages (TAMs) into spermine. Through the p53/thymine DNA glycosylase (TDG)-mediated DNA demethylation pathway, this cascade upregulates peroxisome proliferator-activated receptor γ (PPARG) expression, driving TAMs toward a pro-tumor phenotype, suppressing the anti-tumor activity of CD8^+^ T cells, and ultimately promoting immune escape in BC (Zhu Y. et al., 2025). Inhibition of arginine metabolic enzymes such as argininosuccinate synthase 1 (ASS1) and argininosuccinate lyase (ASL), or degradation of arginine to citrulline by pegylated arginine deiminase (ADI-PEG 20), can deprive tumor cells of exogenous arginine. This strategy selectively kills tumor cells deficient in endogenous arginine synthesis and remodels the immunosuppressive tumor microenvironment (TME) (Yang et al., 2023; Fung et al., 2025). High expression of the arginine transporters SLC7A5 (LAT1) and SLC6A14 in BC suggests that BC cells may actively take up arginine via these transporters to meet metabolic demands (Bai et al., 2025). In a prospective cohort of 170 BC patients treated with doxorubicin, circulating arginine-NO metabolites were analyzed for their association with CTRCD. After doxorubicin treatment, arginine and citrulline levels decreased, while asymmetric dimethylarginine (ADMA) levels increased. As inhibitors of nitric oxide synthase (NOS), elevated ADMA and monomethylarginine (MMA) were significantly associated with an increased risk of CTRCD (Finkelman et al., 2017).

#### H-FABP

H-FABP is a small soluble cytoplasmic protein abundantly expressed in cardiomyocytes, which is rapidly released into the circulation following minor myocardial injury. Yuan et al. (2021) enrolled 19 patients with cancer treated with ICIs and found that H-FABP levels were significantly elevated after 3 months of ICIs therapy, whereas troponin levels remained unchanged. These findings suggest that H-FABP may serve as an early and sensitive biomarker for ICI-induced subclinical myocardial injury, preceding the reduction in LVEF and the onset of clinical symptoms.

#### GPBB

GPBB is an enzyme involved in myocardial energy metabolism and is abundantly released during myocardial ischemia and injury, serving as an early and sensitive biomarker for myocardial ischemia (Cubranic et al., 2025; Krause et al., 1996.). Anthracyclines induce myocardial cell damage by impairing mitochondrial function. A study in acute leukemia patients receiving anthracycline therapy demonstrated that plasma GPBB concentrations were significantly higher at 6 months after treatment than at baseline, and were associated with left ventricular diastolic dysfunction detected by echocardiography. GPBB may represent a potential biomarker for the detection of acute and chronic anthracycline-related cardiotoxicity (Horacek et al., 2013), although its predictive role in BC patients with cardiovascular comorbidities remains to be further investigated.

#### microRNAs

Accumulating evidence indicates that multiple miRNAs form a critical molecular cross-talk network between BC progression and Cancer Therapy-Related Cardiovascular Toxicity (CTR-CVT) by regulating inflammatory responses, oxidative stress, apoptosis, autophagy, mitochondrial function, and endothelial homeostasis. Among these, miR-21-5p is one of the most representative cross-disease regulators in cardio-oncology. In BC, miR-21-5p promotes tumor invasion and metastasis by suppressing tumor suppressor genes such as PDCD4 and TPM1 (Jazbutyte and Thum, 2010), whereas in the cardiovascular system it exerts cell type–dependent effects, participating in myocardial fibrosis, modulation of ischemia–reperfusion injury (Ramanujam et al., 2021; He and Guan, 2025), and regulation of vascular smooth muscle cell proliferation and apoptosis (Zhu et al., 2019), thereby exhibiting pronounced bidirectional regulatory properties.

In DIC, multiple circulating miRNAs have been shown to directly contribute to cardiomyocyte injury. miR-145-5p attenuates DIC damage by suppressing pyroptosis via the SOX9/NLRP3 pathway (Chen X.-T. et al., 2025). miR-34a-5p is markedly upregulated following doxorubicin exposure and promotes cardiomyocyte pyroptosis by targeting Sirt3 and modulating autophagy and mitochondrial reactive oxygen species production, whereas its inhibition confers cardioprotective effects (Zhong et al., 2023). miR-106b-5p exhibits context-dependent effects: it can facilitate tumor progression in BC through PTEN and CNN1 pathways, while in chemotherapy-associated myocardial injury, it exacerbates cardiomyocyte apoptosis and oxidative stress by targeting cardioprotective factors such as FOG2 and sST2 (Asensio Lopez et al., 2025). miR-133a-5p is upregulated in breast cancer patients who develop doxorubicin-related cardiotoxicity and may contribute to myocardial injury via modulation of the ErbB2/HER2 signaling pathway (Alves et al., 2022). Clinical studies further demonstrate that circulating miR-34a-5p, miR-29a-5p, miR-126-5p, miR-499-5p, and miR-423-5p levels are significantly correlated with cardiac troponins (Lakhani et al., 2021), Among these, miR-499-5p and miR-34a-5p exhibit high sensitivity for detecting acute and subacute myocardial injury (Lakhani et al., 2021), whereas miR-126-5p and let-7f are associated with lower cardiotoxicity risk during neoadjuvant chemotherapy in patients with triple-negative BC (Zhu et al., 2018), suggesting potential cardioprotective roles.

Furthermore, exosome-mediated miRNA-driven inter-organ communication has provided novel insights into the pathological BC–heart axis. For instance, tumor-derived exosomal miR-216a-5p induces cardiomyocyte pyroptosis via the ITCH/TXNIP/NLRP3 pathway, constituting a key cross-organ signal driving DIC (Ma et al., 2025c). Conversely, circular RNAs such as circFN1 and circRALGPS2 can act as miRNA sponges, relieving the repression of cardioprotective genes including FOXO3 and ATG7 (Gao et al., 2024), and are therefore considered potential therapeutic targets for mitigating DIC.

In HER2+ BC, miR-130a-5p levels are significantly elevated during chemotherapy and show a positive correlation with declines in left ventricular ejection fraction and increases in cTnI (Feng et al., 2021). Time-series high-throughput analyses have further identified elevated levels of miR-125b-5p, miR-409-3p, miR-15a-5p, miR-423-5p, miR-148a-3p, miR-99a-5p, and miR-320b, as well as reduced levels of miR-642a-5p, as being closely associated with adverse cardiac events, highlighting their potential utility for early prediction of cardiotoxicity during anti-HER2 therapy (Pizzamiglio et al., 2024). In addition, miR-4638-3p and miR-1273g-3p have also been implicated in DIC, with functional enrichment primarily involving cell–cell adhesion, toxic response pathways, and lipid metabolic processes (Yadi et al., 2020).

#### cfDNA

In contrast to microRNA-mediated post-transcriptional regulation, circulating cell-free DNA (cfDNA) primarily reflects downstream signals of cellular injury and death, serving as an important complementary liquid biopsy biomarker for assessing tumor burden and treatment-related tissue damage. cfDNA consists of DNA fragments released into the peripheral blood during cellular apoptosis or necrosis and has emerged as a novel liquid biopsy biomarker capable of dynamically reflecting tumor burden, tissue injury, and therapeutic response. In BC, accumulating evidence indicates that the persistence of circulating tumor DNA (ctDNA) during treatment or after initial therapy is closely associated with an increased risk of recurrence and poor prognosis, and is significantly correlated with tumor size and lymph node involvement (Sant et al., 2022; Beddowes et al., 2025; Dong et al., 2025; Elliott et al., 2025; Garcia-Murillas et al., 2025; Li S. et al., 2025; Lu et al., 2025; Zavarykina et al., 2025).

In patients with HER2+ BC, studies have demonstrated that elevated levels of cardiomyocyte-derived cfDNA following anthracycline-based chemotherapy are associated with an increased risk of CTRCD, suggesting that cardiomyocyte-specific cfDNA may serve as a promising non-invasive biomarker for the early prediction of CTRCD (Yu et al., 2023). TNBC, characterized by the absence of well-defined therapeutic targets and a relatively unfavorable prognosis, poses particular challenges in evaluating response to neoadjuvant immunotherapy and predicting clinical outcomes. Mittendorf et al. (2025), based on the phase III IMpassion031 trial, demonstrated that baseline ctDNA levels and dynamic ctDNA clearance during treatment serve as independent prognostic biomarkers in early-stage TNBC treated with immunotherapy combined with neoadjuvant therapy. Specifically, patients with negative baseline ctDNA exhibited more favorable outcomes, whereas persistent postoperative ctDNA positivity was associated with a markedly increased risk of recurrence, highlighting the potential for treatment adaptation based on ctDNA dynamics. Furthermore, dynamic monitoring of ctDNA, in combination with baseline ctDNA levels and postoperative minimal residual disease (MRD) status to construct tumor burden–based risk stratification models, enables effective prediction of recurrence following neoadjuvant chemotherapy in TNBC, providing a more precise approach for recurrence risk assessment (Li S. et al., 2025). Collectively, dynamic ctDNA monitoring—integrated with baseline burden and postoperative minimal residual disease status—enables refined risk stratification and highlights cfDNA-based assays as a unifying tool for precision oncology and cardio-oncology.

### Imaging diagnosis

Non-invasive imaging modalities complement biomarker-based surveillance, Several observational studies have demonstrated that imaging modalities, such as coronary computed tomography angiography (CTA), cardiac magnetic resonance imaging (CMR), and single-photon emission computed tomography (SPECT) imaging, may also serve as non-invasive assessment options. Correa et al. (2007) reported that in patients receiving radiotherapy for left-sided BC, approximately 85% of coronary artery injuries occurred in the left anterior descending (LAD) artery. Lind et al. (2003) using SPECT imaging, found that all myocardial perfusion defects observed 6 months after radiotherapy in left-sided BC patients were localized to the LAD territory (P < 0.001) and were independently associated with the irradiated volume of the left ventricle. In a comparative observational study, Tu et al. (2024) evaluated coronary CTA in 80 patients treated with anthracycline-based chemotherapy and 59 patients with left-sided BC who underwent anthracycline-based chemotherapy combined with radiotherapy. Post-treatment alterations in computed tomography–derived fractional flow reserve (CT-FFR), pericoronary adipose tissue (PCAT) attenuation, and myocardial extracellular volume (ECV) were consistently observed, suggesting concomitant injury to both coronary arteries and the myocardium. CMR offers superior accuracy in assessing myocardial structure and function, enabling the detection of fibrosis, edema, and subtle early abnormalities, thereby playing a key role in the early identification of myocardial injury.

### Comprehensive diagnosis

Prospective evidence also supports integrated monitoring strategies. In a prospective observational Toronto cohort of 136 women with HER2+ early BC receiving sequential anthracycline and trastuzumab therapy, serial CMR and echocardiographic measures were compared. Using CMR-defined CTRCD as the reference, a stepwise approach with 3D LVEF, 2D GLS, and 2D GCS improved diagnostic accuracy compared with single measures. In contrast, the serum biomarkers such as hsTn and BNP failed to add predictive value due to insufficient sensitivity and weak correlation with the CMR-based diagnostic criteria (Esmaeilzadeh et al., 2022). The findings of this study suggest whether this method can be used to detect subclinical cardiac dysfunction at an early stage and enable intervention before irreversible damage occurs, thereby ensuring the treatment safety and long-term quality of life of cancer patients. Furthermore, in asymptomatic patients with preserved cardiac function who have been exposed to cardiotoxic agents, structured surveillance is recommended. Current evidence supports reassessment using cardiac biomarkers and imaging. A combined strategy that integrates biomarkers and advanced imaging offers the best opportunity to detect subclinical dysfunction early.

## Treatment

For patients with BC combined with CVD, treatment needs to follow the principle of integrated treatment, taking into account the management of BC and CVD.

### Lifestyle interventions

#### Dietary modification

Several observational studies have demonstrated that under chemotherapy-induced oxidative stress and inflammation, many dietary nutrients with cardioprotective properties have garnered attention. Polyphenols, a natural antioxidant, are widely found in fresh fruits and vegetables. Studies have shown that polyphenols can inhibit transcription factors like NF-κB and other tumor-promoting proteins, thereby reducing BC cell proliferation and metastasis (Bhattacharya et al., 2021; Messeha et al., 2021). Also, polyphenols suppress aromatase activity and estrogen production, antagonizing estrogen receptor signaling (Cipolletti et al., 2018; Nielsen and McNulty, 2019). Other antioxidants such as ginseng, alpha—lipoic acid, vitamin D, and vitamin E have shown a potential to neutralize ROS. Coenzyme Q10 (CoQ10), found in foods such as fish, meat, vegetables, fruits, nuts, and oils, helps stabilize mitochondrial function and reduce oxidative damage to cardiomyocytes during chemotherapy (Al-Hammadi et al., 2023). Findings point to higher vitamin D levels being associated with substantial reductions in CVD incidents, T2DM, and metabolic abnormalities (Giandalia et al., 2021). Nutritional supplementation with vitamin D and ginseng may help regulate inflammation, improve lipid profile, and is essential for maintaining cardiovascular health in patients undergoing chemotherapy for BC.

#### Physical exercise

In patients with BC combined with CVD, physical exercise can effectively lower pro-inflammatory biomarkers and reduce CVD risk factors (Campbell et al., 2019; Chan et al., 2019), As an important component of physical exercise, several randomized controlled trials (RCTs) have demonstrated that aerobic exercise reduces serum cholesterol levels, decreases cholesterol uptake by tumor cells, enhances the body’s anti-tumor immune response, and lowers the risk of CVD (Gonzalo-Encabo et al., 2023; Al-Mhanna et al., 2024), high-intensity interval training (HIIT) is a training method that alternates high-intensity exercise with short rest. Compared with continuous aerobic exercise, HIIT can increase blood flow and insulin receptor expression, and upregulate GLUT-4 in skeletal muscle, thereby improving insulin resistance (Christ-Roberts et al., 2004). Gonzalo-Encabo et al. (2023) found that compared with the control group, BC patients receiving 8 weeks of HIIT significantly reduced blood glucose, lipids, CRP, leptin, and other markers of BC patients treated with anthracyclines, alleviating the series of adverse consequences triggered by BC treatment. Nonetheless, the effect of physical activity on this disease still needs to be further confirmed by more clinical trials.

### Pharmacologic therapy

Given that treatments such as radiotherapy and chemotherapy for BC can cause myocardial damage, drug selection must be cautious in patients with pre-existing CVD. In terms of chemotherapy drug selection, liposomal anthracyclines combined with cardioprotective agents such as dexrazoxane are commonly recommended to reduce oxidative stress injury and minimize cardiotoxicity (De Baat et al., 2022). Furthermore, considering the prothrombotic state exacerbated by chemotherapy-induced endothelial injury, individualized anticoagulation strategies—utilizing low-molecular-weight heparin (LMWH) or direct oral anticoagulants (DOACs)—are essential to prevent cancer-associated thrombosis (CAT) (Mahé et al., 2025). Patients receiving targeted therapies require rigorous cardiac assessment; in high-risk cases, dose adjustment or combination therapies should be considered.

For perimenopausal women, endocrine therapy requires careful selection. Tamoxifen, for instance, can lead to dyslipidemia and endometrial hyperplasia, thereby increasing the risk of CVD and endometrial cancer (Alomar et al., 2022; Ryu et al., 2022). Importantly, Tamoxifen is also associated with a significantly higher risk of VTE compared to AIs (Xu et al., 2019). While AIs may be an alternative, their potential to cause bone loss and joint pain must be addressed (Hadji et al., 2017).

In terms of metabolic modulation, selected agents should ideally offer both glycemic control and cardioprotection. Evidence-based therapies include ACEi, ARBs, aldosterone receptor antagonists (MRAs), SGLT2 inhibitors (SGLT2i), GLP-1 receptor agonists (GLP-1RAs), and statins. As discussed earlier, systematic reviews and meta-analyses, RAAS-targeting drugs may mitigate long-term heart failure risks induced by anthracyclines or trastuzumab (Li et al., 2020; Livi et al., 2021; Mir et al., 2023). Both preclinical studies and randomized controlled trials have confirmed that SGLT2i and GLP-1RA, as novel hypoglycemic agents, significantly reduce key cardiovascular and renal endpoints (Wiviott et al., 2019; Hundal et al., 2025). In BC, they inhibit renal glucose reabsorption, suppress tumor glycolysis and ATP production, and reverse hyperinsulinemia to slow tumor growth (Scafoglio et al., 2015; Koepsell, 2017; Nasiri et al., 2019; Komatsu et al., 2020; Zhou et al., 2020b). Additionally, SGLT2i reduce albuminuria, inhibit the IL-βinflammatory pathway, and synergize with the RAAS to minimize fluid retention (Fonseca-Correa and Correa-Rotter, 2021). Statins, beyond lipid regulation, may counteract DIC by inhibiting Rho GTPases and reducing ROS production (Li et al., 2020). Collectively, the integration of cardioprotective, metabolic, and anticoagulant therapies establishes a comprehensive pharmacological framework to safeguard the systemic health of BC patients.

### Radiation therapy

With the adoption of advanced radiotherapy techniques—including intensity-modulated radiation therapy (IMRT), proton therapy, prone positioning, partial breast irradiation, and deep inspiration breath-hold (DIBH)—the risk of radiation-induced heart injury (RIHD) has been substantially reduced. IMPT can minimize damage to surrounding normal tissues while highly focusing radiation energy on the tumor target, significantly reducing the dose to organs at risk and lowering the risk of adverse cardiac effects, making it particularly suitable for left-sided BC and mediastinal tumors adjacent to the heart (Cao et al., 2025b). Nonetheless, RIHD remains a clinical concern due to its long latency period, low specificity of clinical manifestations, and the presence of multiple confounding factors, all of which complicate its diagnosis and risk stratification. Current evidence supports minimizing cardiac exposure, with the RTOG 1304 clincial trial suggesting that the mean heart dose (Dmean) should be kept below 4 Gy during whole-breast or chest wall irradiation for patients with left-sided BC (Mamounas et al., 2025). However, uniform dose constraints for the coronary arteries have yet to be established. Furthermore, it remains unclear which specific cardiac substructures are most susceptible to radiation damage and can be reliably used as reference indices for cardiac tolerance. Determining such critical thresholds and formulating preventive standards will require robust evidence from future large-scale clinical studies.

### Emerging therapeutic interventions

The exploration and translation of emerging intervention strategies have provided patients with more diversified therapeutic options. Zhang F.-S. et al. (2026) reported that the anti–BC agent Neratinib exerts pronounced anti-vascular inflammatory and anti-atherosclerotic effects. Notably, these anti-inflammatory actions are independent of its canonical HER2-targeting activity and are instead mediated through inhibition of the ASK1/JNK/p38 signaling pathway, thereby effectively suppressing inflammatory signal transduction in endothelial cells. Further studies demonstrated that the combined administration of lapatinib with the widely used lipid-lowering agent rosuvastatin not only preserves lipid-modifying efficacy but also exerts additive suppression of vascular inflammation, resulting in a more pronounced attenuation of atherosclerotic progression.

At the molecular level, bidirectional CRISPR interference/activation (CRISPRi/a)–based high-throughput screening in human iPSC-derived cardiomyocytes (iCMs) revealed that doxorubicin aggravates cardiomyocyte glycolysis through activation of carbonic anhydrase 12 (CA12), thereby disrupting metabolic homeostasis and inducing functional impairment. Pharmacological inhibition of CA12 with indisulam was shown to mitigate anthracycline-induced glycolytic dysregulation in cardiomyocytes, identifying CA12 as a potential therapeutic target for chemotherapy-related cardiotoxicity (Liu C. et al., 2024).

In parallel, advances in targeted nanomedicine have increasingly focused on tumor microenvironment–responsive and cardioprotective co-design strategies. A variety of nanocarriers, including liposomes, polymeric nanoparticles, polymeric micelles, dendrimers, carbon nanotubes, zinc–copper metal nanoplatforms (CZP NPs), manganese dioxide nanoparticles (MnO_2_NPs), and sonosensitizing nanoshuttle systems (DPPM@HA), have demonstrated the capacity to preferentially accumulate within BC tissues, enabling precise tumor-targeted drug delivery. These platforms achieve effective antitumor activity while substantially reducing cardiac toxicity, thereby offering promising and safer therapeutic alternatives for patients with BC complicated by CVD (Bhandari et al., 2025; Zhou et al., 2025; Yu et al., 2025; Zhang S.-M. et al., 2026).

## Conclusion

In the complex field of BC combined with CVD, milestones have been achieved in lifestyle intervention, pharmacologic therapy, functional monitoring, and multidisciplinary collaboration. Nevertheless, in the early stage of the disease, due to the insidious symptoms and lack of typical manifestations in early stages, such comorbidities are frequently underdiagnosed, delaying timely intervention. This underscores the need for a standardized cardiovascular monitoring framework that incorporates baseline cardiac status and treatment-specific risks to guide individualized strategies. Such protocols should routinely include structural indices such as left ventricular ejection fraction (e.g., echocardiography every 3 months during HER2-targeted therapy), as well as biochemical markers including troponin and NT-proBNP. In parallel, digital health technologies—such as wearable devices capable of tracking heart rate, blood pressure, and oxygen saturation—can enable continuous data collection and dynamic alerts, thereby complementing periodic assessments and enhancing the timeliness and accuracy of early risk detection.

Mounting evidence has highlighted the critical roles of metabolomic characteristics and interrelated biological processes in mediating the complex crosstalk between BC and cardiovascular dysfunction. Application of the single-cell metabolomics platform ID-organic cytoMS has revealed metabolic heterogeneity of β-hydroxybutyrate (BHB) in MCF-7 BC cells. BHB was found to modulate oxidative stress responses via upregulation of MT2A, with its abundance linked to BC sub-type differentiation (Qin et al., 2024). These findings provide a rationale for targeting oxidative stress pathways and highlight the potential of metabolic interventions. Indeed, LC-MS–based metabolomics has proven effective in differentiating metabolic profiles between BC patients and healthy individuals, eleven prospective nested case-control metabolome-wide association studies (MWAS) have identified 41 candidate biomarkers associated with BC risk. However, merely five metabolites have been reported to be significantly modulated in more than one study (Füreder et al., 2025), By delineating the associations between metabolite abundance and disease subtypes, it may be possible to optimize metabolic regulation, balancing glycemic control with tumor metabolism and cardiovascular protection—achieving a synergistic strategy of “metabolic management–tumor control–cardiovascular protection.

In recent years, emerging biological processes such as epigenetic alterations, clonal hematopoiesis, and gut microbiota dysbiosis have been increasingly recognized as potential mechanistic links between cancer and CVD (Chen C. et al., 2025; Hundal et al., 2025; Liu H. et al., 2025; Wang et al., 2025d; Xue M. et al., 2025; Delzenne et al., 2025). These pathways may contribute not only to long-term cardiovascular risk in cancer survivors but also to the substantial inter-individual heterogeneity observed in cardiovascular outcomes following cancer diagnosis and treatment. Growing evidence suggests that cardiovascular risk in cardio-oncology is not static, but dynamically evolves with tumor progression, treatment exposure, and host-related factors. Accordingly, future research should move beyond static association analyses and adopt more mechanism-informed and longitudinal perspectives. The integration of molecular profiling approaches with dynamic clinical indicators—including biomarkers, imaging features, and circulating tumor-related measures—may help to better characterize shared inflammatory, metabolic, and regulatory pathways linking BC and CVD, ultimately improving cardiovascular risk stratification and individualized prevention strategies.

It should be acknowledged that, despite substantial progress in mechanistic research and risk assessment within the field of cardio-oncology, the translation of these advances into routine clinical practice remains challenged by several practical constraints. At present, the accessibility and cost of multi-omics testing, novel therapeutic agents, and advanced imaging modalities vary considerably across healthcare settings, underscoring the need for careful evaluation of their appropriate use within evidence-based frameworks. Moreover, risk assessment and management strategies for CTR-CVT have not yet been fully standardized, with persistent heterogeneity in clinical pathways and practice patterns across regions and institutions, highlighting the need for further refinement of clinical guidelines and operational protocols. In addition, long-term follow-up remains difficult to implement in many settings, owing to suboptimal patient adherence, uneven allocation of follow-up resources, and challenges in cross-center data integration, which collectively limit comprehensive evaluation of long-term cardiovascular outcomes. Future studies should therefore balance continued advances in mechanistic understanding with greater attention to clinical feasibility and implementation, promoting standardized care pathways, optimized follow-up strategies, and strengthened multicenter collaboration to facilitate the gradual translation of research findings into sustainable and broadly applicable clinical practice, while ensuring long-term cardiovascular safety alongside effective cancer control.
